# Healthy human gut microbiome: Towards standardized research

**DOI:** 10.3934/microbiol.2025034

**Published:** 2025-11-04

**Authors:** Evgeniya Glazunova, Polina Molodtsova, Ilya Grabarnik, Alexander Kurnosov, Irina Bikaeva, German Shipulin, Olga Zlobovskaya

**Affiliations:** 1 Federal State Budgetary Institution “Centre for Strategic Planning and Management of Biomedical Health Risks” of the Federal Medical and Biological Agency, Russia; 2 Applied Genomics Laboratory, ITMO University, Saint Petersburg, 197101 Russia; 3 N.M. Emmanuel Institute of Biochemical Physics of the Russian Academy of Sciences, Moscow, 119334 Russia

**Keywords:** human gastrointestinal microbiota, fecal microbiome, normal gut microbiome, sample homogenization, bead beating, 16S rRNA gene sequencing

## Abstract

**Objective:**

An increasing number of international researchers are focusing on the taxonomic composition of fecal microbiota and its correlation with disorders. Thousands of researchers compare conditionally healthy cohorts to those with specific diseases to identify potential markers. However, clinical application requires assessing the feasibility of synthesizing these findings and establishing reference intervals for normal gut flora, at least at higher taxonomic levels.

**Design:**

This study involves a systematic review and meta-analysis of human gut microbiota research based on 16S rRNA gene next-generation sequencing (NGS). Relevant research was sourced following the PRISMA guidelines. Descriptive statistics, linear regression analysis by weighted least squares method, Mann-Whitney test, and Benjamini-Hochberg procedure adjustments were employed. The study has been registered with PROSPERO (CRD42023431467).

**Results:**

Of the 4,346 studies initially identified, 86 publications involving 20,748 unique participants met the quality criteria and were included in the analysis of the impact of fecal sample preparation on taxonomic composition. The phylotype composition, in relation to preprocessing methods and cohort locations, are presented as relative abundances (%): Bacillota (median 49.5–59.6%), Bacteroidota (28.0–33.4%), Pseudomonadota (3.4–5.9%), Actinomycetota (2.3–3.7%), Verrucomicrobiota (0.5–1.0%), Fusobacteriota (maximum 4.6%), and Euryarchaeota (maximum 2.11%). The content of 27 key family-level representatives was also evaluated. The well-known hypothesis regarding the influence of the homogenization stage on taxonomic composition was examined using generalized results.

**Conclusion:**

While supported by a strong theoretical basis and evidence from individual practical cases, none of the phyla showed a statistically significant association and consistent relationship with sample preparation or cohort location when generalizing across studies after the two exceptionally large cohorts exclusion, both originating from a single research group. These findings underscore the need for strict methodological standardization in microbiome studies. Key features of the 16S NGS process accounting for these results are outlined, along with proposed optimizations for microbiome research.

## 1. Introduction

Since the 2000s, the diversity of microorganisms within the human gastrointestinal tract, collectively termed the microbiota, has been intensely investigated. Numerous publications from different countries evaluate the influence of the intestinal microbiota on the host organism and its associations with functional disorders or conditionally healthy state. Rapidly advancing molecular biological methods for studying the host-microbiome interaction, based on metagenomic analysis, facilitate the qualitative and quantitative determination of the taxonomic groups of the microbiome community extracted directly from the investigated ecosystem. Major foundational sequence-based studies of the human gut microbiome include the Human Microbiome Project, funded by the US National Institutes of Health [1],[2]; the Metagenomics of the Human Intestinal Tract (MetaHIT) project [3], funded by the European Commission; and the American Gut Project [4], an ethnic study, along with a prospective general population cohort study in the Netherlands (LifeLines DEEP) [5].

The outer mucosal layer of the human gastrointestinal tract is inhabited by a large, complex, and highly diverse microbial community, comprising between 10 and 10^2^ trillion microorganisms represented by various species. The microbiota acts as a constant symbiont of humans throughout life and plays an active role in numerous biological processes [6]–[8]. While their functions are unified, the composition of individual microbial communities exhibits significant variation, particularly at the family and genus levels [9],[10].

**Figure 1.**
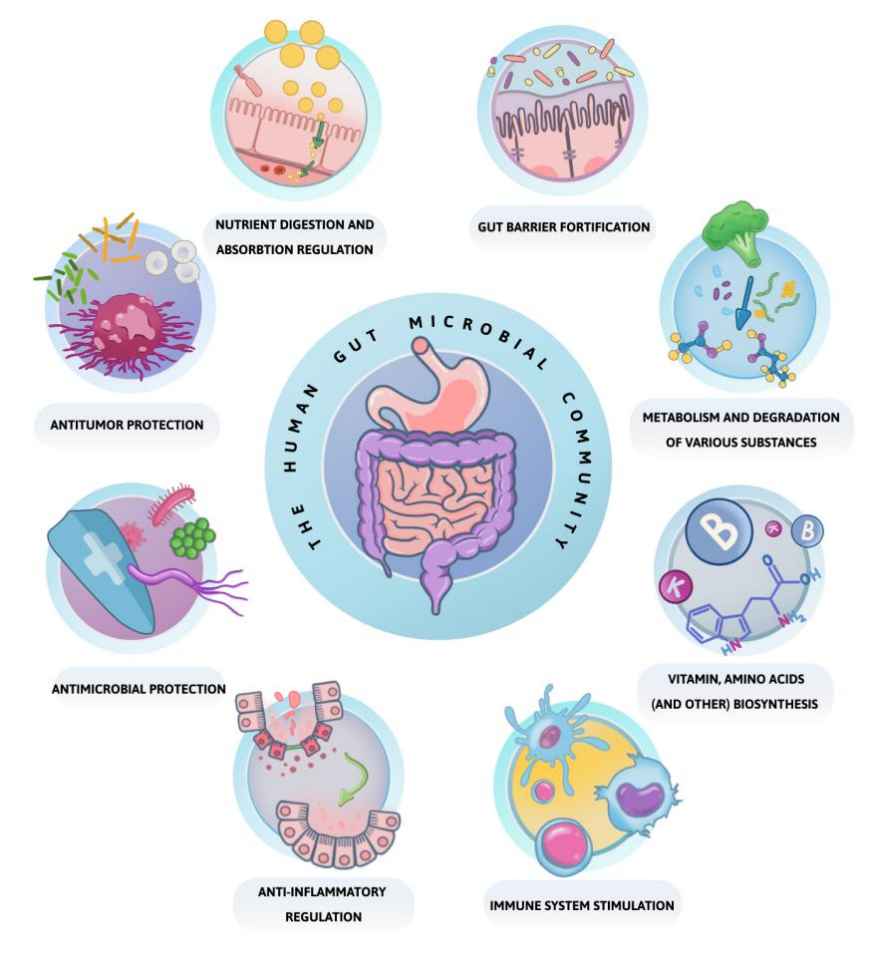
The human gut microbial community fundamental functions.

The human gut microbial community is primarily represented by two major domains of life: Bacteria and Archaea [6],[8],[11]–[15]. The gut microbiota is increasingly recognized as a functionally integrated part of the human body, effectively functioning as an organ. The crucial roles of intestinal microbiota include direct and indirect contributions to the regulation of digestion [11],[16]; extraction, absorption, and production of various substances, including those that nourish intestinal epithelial cells [11],[16]–[18], and exhibit probiotic activity [11],[19]. The microbiota also contributes to increased regulatory T cells memory [15],[17],[20], T cell growth, and proliferation [18],[20]–[22]; antitumor and anti-inflammatory processes [15],[18],[21],[23]; and the expression of antimicrobial peptides [22]. Furthermore, it fortifies the epithelial barrier by normalizing mucosal layer thickness [10],[24],[25], stimulating colonocyte growth [18],[26], enhancing the expression of tight junction protein genes [23], and suppressing pathogenic bacterial growth [10],[27],[28] (see Figure 1).

The intestinal symbiotic community is shaped by early-life colonization and age-associated changes. However, in healthy adult subjects, the ratio of major taxa remains relatively stable [8],[10],[11],[14],[27]. A normal intestinal host-microbiome is predominantly represented by facultative and obligate (strict) anaerobes. Key players include Bacteroidota [Bacteroidetes] and Bacillota [Firmicutes] phyla, which account for more than 90% of all bacteria, while Actinomycetota [Actinobacteria] [29], Pseudomonadota [Proteobacteria], and Verrucomicrobiota [Verrucomicrobia] are common but generally minor constituents [7],[10],[11].

It is important to reiterate, however, that individual gut communities are unique, differing qualitatively and quantitatively from person to person and influenced by factors such as age, region of residence, lifestyle, and diet [27],[30]. For instance, many studies indicate that the ratio of the dominant taxa, Bacillota [Firmicutes] and Bacteroidota [Bacteroidetes] (F/B ratio), in healthy subjects is dependent on dietary intake. In particular, the microbiome of vegetarians differs from that of individuals adhering to the “Western” diet: An increase in the relative abundance of Bacillota bacteria is associated with a high-fiber diet, while an increase in Bacteroidota correlates with a low-fiber diet [7],[10],[15],[18].

Data on the structure of the human gut microbiota are most often obtained through various methods of fecal material analysis due to its availability, microbial enrichment, and the non-invasive nature of sample collection.

Bacterial cultivation on selective media has long been a classical method for studying the human microbiota. With advancements in microbiological techniques, initial knowledge regarding the microbial community has accumulated. These techniques continue to be widely used in clinical practice for diagnostic research of microbial communities. However, they cannot be fully applied for qualitative analysis, and particularly quantitative analysis, of complex systems such as the gut microbiome. Traditional culture methods only successfully cultivate approximately 10% of gut microbiota species and are highly dependent on the quality of material collection and storage [2],[8].

Methods based on the analysis of microbial DNA directly isolated from samples offer a variety of significant important advantages over cultivation techniques. These include the ability to detect a wide range of non-cultivable species, reduced dependence on collection and storage conditions, and the capacity to estimate the relative abundance of taxa within the sample. Significant progress in the investigation of microbiota composition has occurred with the widespread availability of one DNA amplification method: PCR. PCR is a simple, sensitive, and highly specific method. The advent of the real-time PCR method in 1993, enabling amplification with simultaneous visualization of product accumulation, has enabled fast qualitative and, importantly, quantitative analysis of microbial communities.

Over the past decade, with the rapid advancement of molecular technologies such as various next-generation sequencing methods, a substantial amount of data on gut microbiome composition has been generated. Most of these data are based on the amplification of 16S ribosomal rRNA gene regions followed by sequencing.

16S rRNA sequencing is a widely utilized method for studying the diversity of microbiota taxa. However, this technology relies on only one genomic region and is therefore inadequate for a comprehensive analysis of the microbiome, as well as for assessing the gut community's detached functions and interactions. For this purpose, a more time-consuming and costly approach is employed: High-throughput sequencing with total genome-based taxonomic identification. Such metagenomic analysis enables researchers to study the taxonomic composition and abundance of microbiome components, discover new species, and track changes in community dynamics. However, this analysis is labor-intensive and expensive, particularly concerning information processing and data interpretation.

**Figure 2.**
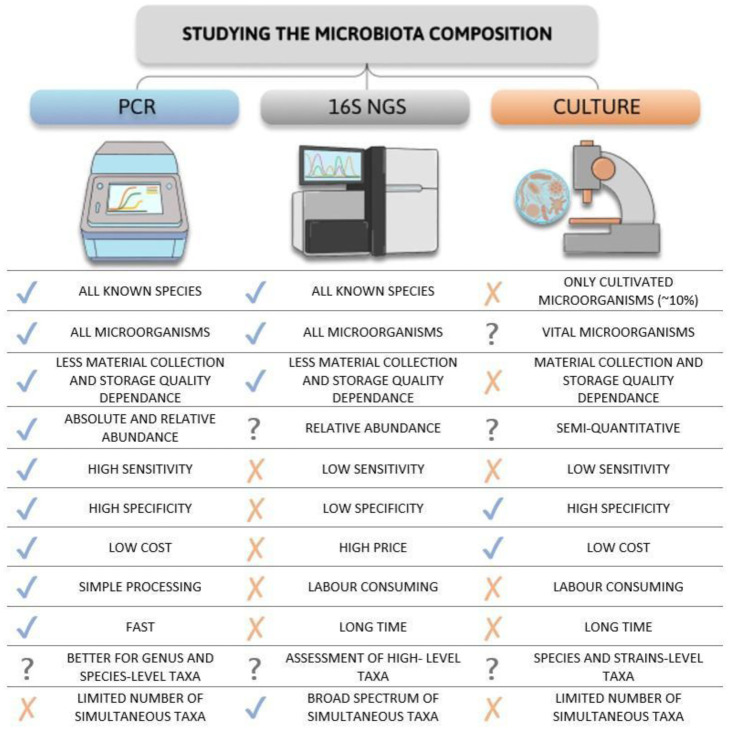
Comparison of the advantages and drawbacks of qPCR, NGS, and bacterial culture methods.

The benefits and disadvantages of the described analytical methods are illustrated in Figure 2.

When studying the microbiome structure using molecular biology techniques, it is essential to consider various methodological aspects, such as the DNA isolation technique. qPCR and, particularly, NGS methods are heavily dependent on the quality of the applied DNA extraction technique. Published studies have shown that fecal mechanical homogenization (“bead beating”) positively impacts the overall efficiency of DNA isolation, especially from Gram-positive bacteria [31]–[33]. However, despite the availability of the International Human Microbiome Standards (IHMS) [34] and recommendations outlined in IHMS SOP 06 and IHMS SOP 07 [35],[36] concerning material collection, storage, and DNA extraction, protocols vary significantly across many published studies [12],[32],[37]–[50].

A critical question arises regarding the reliability of synthesizing data from different studies to draw meaningful conclusions. Even with a substantial evidence base from primary sequence-based studies of the human gut microbiome, individual reports yield varying conclusions regarding the relative abundance percentages of the principal microbiome representatives. The primary objectives of this systematic review and meta-analysis are to taxonomically describe the human fecal microbiome in healthy subjects analyzed using 16S NGS. We also aim to investigate the correlation between sample preparation methods, specifically the presence or absence of a mechanical homogenization step, and the resulting sequencing data. Together, these analyses aim to assess whether existing literature enables the establishment of reliable reference intervals for taxa of the normal gut microbiome for future applications. This issue holds considerable medical and economic significance, particularly as many laboratories have recently begun offering services to determine the composition of intestinal flora and provide subsequent recommendations regarding lifestyle modifications and even “cures”.

## 2. Materials and methods

### 2.1. Search strategy and selection criteria

**Figure 3.**
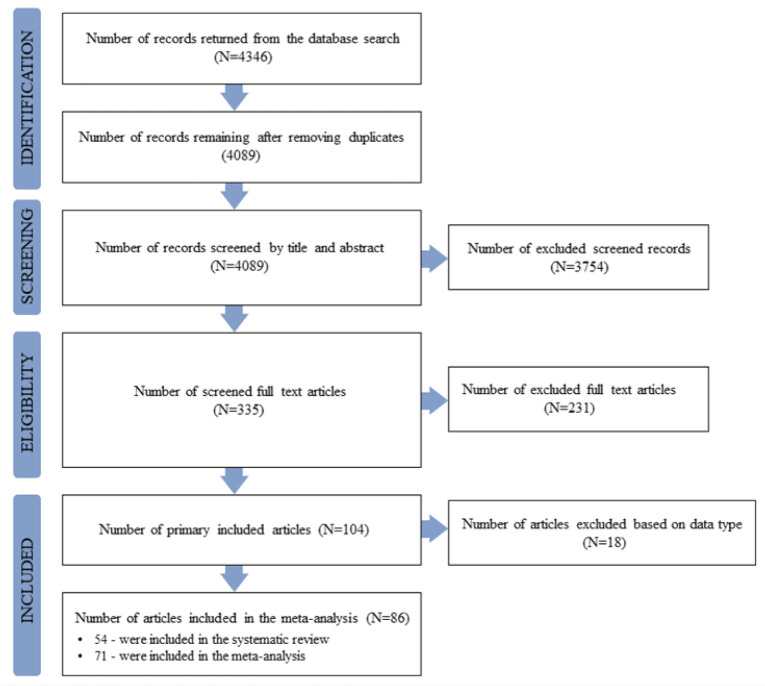
PRISMA flow chart.

For this systematic review and meta-analysis, we conducted an article search strategy, data collection, and analysis in accordance with PRISMA statement guidelines [51], utilizing a pre-selected search strategy based on our inclusion and exclusion criteria. A PRISMA flow chart detailing the search strategy is presented in Figure 3. Our protocol was pre-registered with PROSPERO (CRD42023431467).

### 2.2. Searching process and article number at each selection stage

The search was conducted between November 2022 and May 2025, encompassing studies published since 2000 regardless of language. Three experts independently performed the screening of publications, removal of duplicates (via link screening), selection, analysis, and data extraction. Disagreements regarding inclusion were resolved through consensus among all authors of this review.

The meta-analysis included studies from the NCBI [52] database, focusing on human intestinal microbiota composition across various study designs, including fundamental research, randomized controlled trials, case-control studies, cohort studies, and reviews. References to relevant data presented in the reviewed publications were also assessed. Our search queries, which incorporate well-established terms characterizing the microbiome, materials, and research results, are provided in Supplementary materials. The literature search strategy, including all keywords and operators is presented in Supplementary materials.

Next, we screened the titles and abstracts of all selected full-text articles, choosing those that matched the search topic and eliminating duplicates. Following this, during the eligibility stage, we reviewed and selected full-text articles based on our inclusion/exclusion criteria (see Supplementary materials).

### 2.3. Quality and risk of bias of individual studies

As this is a systematic review and meta-analysis entailed the microbiome in a healthy state, and given that the included studies addressed diverse objectives, a direct assessment of study outcomes was not appropriate. Our research question was based solely on the baseline, control, or placebo groups reported in the included publications. Therefore, rather than evaluating individual study effects on overall results, the eligibility of the healthy subject selection across investigations was assessed according to recommended guidelines based on the declared study design.

1) To assess the risk of bias for research studies (including case-control and cohort studies), we evaluated the data using the following criteria from the Newcastle-Ottawa scale [53]:

 a) Category “Group Selection”—paragraphs 3 and 4

 b) Category “Comparability”

 c) Category “Exposure” (for case-control studies)

 d) Category “Cohort Selection” (for cohort studies)—paragraphs 2, 3, 4, and 5.

2) For pilot studies, such as randomized controlled trials (RCTs), the data were evaluated according to the methodology based on the Cochrane Community Guidelines [54].

3) For systematic reviews and meta-analyses, we used the AMSTAR (A Measurement Tool to Assess Systematic Reviews) methodology [55] to check the data.

4) For fundamental research, we conducted quartile-based evaluations of the journals during the period of article publication [56].

To assess the risk of diagnostic errors in the formation of study groups (including healthy participants), we analyzed the diagnostic methods utilized by researchers. We posited that the more comprehensive the medical examination, the less likely the participants were to be misdiagnosed as “healthy.” We rated the included studies based on the diagnostic methods employed: The higher the score, the greater the potential error rate (Tables S1 and S2).

While the exclusion criteria for participants varied among studies, we also considered the specified individual exclusion criteria utilized to assess the health status of subjects and to form a representative selection. Additional exclusion criteria are provided in Tables S3–S6.

### 2.4. Data analysis

We extracted data from the evaluated and selected publications, including bibliographic details like the first author, year of publication, and journal SJR. We also collected information about the study parameters, such as the aim, design, and additional selection criteria for healthy subjects. Participant characteristics, including the number of participants, sex, age, cohort location, cohort name, and study population, were recorded. Additionally, we noted methodological details, such as the biomaterial collection process, DNA extraction method, and 16S rRNA target region. Finally, we extracted results on the relative abundance of bacterial and archaeal phyla and families, all expressed as percentages at the level of summary estimates.

Since most of the presented data were expressed as means, studies reporting data as medians were subsequently excluded from the meta-analysis.

If the article reported healthy participants divided into subgroups labeled “baseline or control group” and “placebo”, both groups were included and analyzed as independent cohorts. In studies presenting “before and after placebo” data related to a single group of subjects, such data were averaged for that group.

### 2.5. Statistical analysis

The extracted raw data concerning the qualitative composition of the microbiome and the most significant representatives (phyla and families), expressed as relative abundance percentages, were grouped and subsequently analyzed according to the DNA extraction method and cohort location.

Taxa were included in the meta-analysis only if data on their relative abundance were reported in at least eight studies (for phyla) or seven studies (for families) for the compared groups; otherwise, they were considered only in the systematic review.

Statistical analysis of the relative abundance of key bacterial and archaeal taxa (including weighted averages, value dispersion, medians and quartiles calculations) as well as their ratios was performed using the SciPy v.1.9.2 and Statsmodel v.0.14.0 packages for Python.

Paired group-level means of taxa relative abundance (or their ratios) were analyzed using weighted least squares (WLS) regression, with the DNA extraction method and cohort location as explanatory variables and weights proportional to the number of observations in each study. The choice of WLS was driven by its capacity to address variance heterogeneity and variations in sample sizes observed in the studied data. Unlike non-parametric methods, which are robust to distributional violations but lack flexibility in modeling complex dependencies or directly adjusting for unequal variances, WLS offers a parametric framework that corrects these issues through weights proportions and enhances the precision and reliability of the analysis.

To account for sampling heterogeneity, we performed parallel analyses based on two datasets. The complete dataset included two exceptionally large studies (n = 893 and n = 582) conducted by the same research group, which exceeded the mean sample size by 23- and 15-fold respectively, and strongly influenced the statistical significance. Results for both the complete dataset and the balanced subset excluding these studies are presented.

We estimated the appropriate parameters of the Box-Cox transformation, which was applied to the relative abundances and the ratios data, in order to stabilize the variance and approach data to a normal distribution. This transformation enabled us to meet the assumptions of the Gauss-Markov theorem: Random distribution of residuals, zero mean of residuals, homoscedasticity of residuals (assessed using the Breusch-Pagan test via statsmodels.stats.diagnostic.het_breuschpagan), absence of autocorrelation in residuals (assessed using the Durbin-Watson test via statsmodels.stats.stattools.durbin_watson), and normality of residual distribution (assessed using the Shapiro-Wilk test via scipy.stats.shapiro). Given the multiple comparisons across taxa, the Benjamini-Hochberg (FDR-BH) correction was applied with the significance level (alpha) set at 0.05.

The non-parametric Mann-Whitney U test with FDR-BH correction was applied to additionally assess and verify the statistical differences between the abundance of all groups, especially those that did not meet any of the listed assumptions.

However, the calculation of weighted averages or other similar parameters, alongside their statistical analysis, was not feasible for taxa with limited amount of data (less than eight studies for phyla or seven studies for families). To assess the structure and diversity of the microbial community for these taxa, we listed the individual relative abundance values of bacteria and archaea.

## 3. Results

### 3.1. Study selection and characteristics

We searched the NCBI database for studies that have been published since 2000, focusing on the intestinal core microbial community of conditionally healthy adult donors. Research was conducted between November 2022 and May 2025, employing well-established search terms that characterize the microbiome and keywords for major taxonomic representatives (at both phylum and family ranks), in accordance with PRISMA statement guidelines. Key exclusion criteria for submitted data included insufficient methodological descriptions and a total percentage of identified bacterial and archaeal taxa less than 90%. Articles were not restricted by study design, language, or donor location. Included studies provided descriptions of the selection of healthy subjects, including adequate declarations of excluded diseases, medication intake, abnormal conditions, or special lifestyle and dietary habits.

Among 4,346 studies published, 4,089 unique reports were identified (see Figure 3). 3,754 records were excluded from full-text assessment following title and abstract screening. A total of 335 full-text articles were screened for eligibility; 231 publications were excluded for not meeting all eligibility criteria (see the “Inclusion and Exclusion Criteria” in the “Searching Process and Article Number at Each Selection Stage” section).

Of the 104 articles, 86 contained data expressed as means, 15 presented data as medians, 1 did not report any statistical parameters for the published data, 1 did not report inclusion/exclusion criteria for controls, and 1 did not identify the phylum Bacteroidetes, resulting in its exclusion from the meta-analysis (see Table S7). The analysis included 86 studies based on 20,748 unique, healthy adult participants. A total of 71 of these studies contained data on the representation of dominant bacterial and archaeal phyla and were included in the meta-analysis, while 54 provided data on family diversity for the systematic review.

For this systematic review, the included studies were classified into two groups: Those with and those without preliminary sample mechanical homogenization (bead beating). Summary of studies and participants characteristics is in the Tables S3–S6. Among the 86 studies included in this analysis (which summarize study and participant characteristics and basic information on participants in Tables S3–S6), 54 reported pre-homogenization methods (see Tables S3 and S4), while 32 articles used protocols that excluded this step (see Table S5 and S6), resulting in 57 and 26 samples, respectively.

A significant proportion of the included studies involved participants from various regions in China (n = 27) and from different regions of Europe (n = 24). Other countries in East Asia (Japan, Taiwan, and South Korea) contributed ten publications, and Mongolia contributed one; several Southeast Asia countries (Singapore, Malaysia, Indonesia, and Thailand) accounted for five publications; and South Asia (India and Pakistan) was represented by five publications. European countries collectively accounted for 24 publications. In particular, Western and Northern European countries (United Kingdom, Netherlands, Denmark, France, Belgium, and Sweden)/Southern European countries (Italy and Spain) were each represented in ten publications; and Central and Eastern European countries (Poland, Russia, and Slovenia) were represented by four articles. Participants from North America (the United States and Mexico) contributed nine publications; studies from South America (Argentina and Brazil) encompassed three publications; and Countries of Oceania (New Zealand and Australia) each contributed one publication.

We formed four subgroups based on the geographical location of the participants and presence of the bead beating stage, considering that the number of relevant studies available for comparison was similar within the groups with and without the homogenization stage. These subgroups were the “Eastern subgroup” (comprising 31 samples with pre-homogenization), likely following a traditional plant-based diet characterized by a predominance of complex fibers, and the “Western subgroup” (comprising 26 samples with pre-homogenization), associated with a Westernized diet predominantly featuring animal proteins. Within the group without bead beating, there were 16 samples from “Eastern subgroup” donors and ten samples from “Western subgroup” donors.

### 3.2. Correlations of key representatives of the normal human gut at the phylum and family levels with the sample preparation method: Generalized results show weak correlation with one-study quantitative findings

Since the sample sizes varied substantially and the data distributions deviated from normality (a particularly critical factor when the group sizes differ) to compare the two groups based on the transformed data, the weighted least squares method was applied. Following Box-Cox transformation, the data for most taxa groups largely satisfied the assumptions of the Gauss-Markov theorem, i.e., observed residuals were not systematically skewed, had near zero means, and exhibited normality, minimal autocorrelation, and homoscedasticity. However, certain taxa demonstrated deviations, particularly in terms of variance homogeneity and residuals normality (Firmicutes, Bacteroides, Verrucomicrobia, and Cyanobacteria phyla each failed at least one of the applied tests). Despite these exceptions, applying WLS to the full dataset remains acceptable, as diagnostic tests failures primarily indicate suboptimal efficiency of ordinary least squares estimation rather than methodological invalidity, whereas the used method is robust to mild heteroscedasticity when appropriate weights are applied, while excluding affected taxa could reduce representativeness.

We identified representatives of 16 major phyla and 27 significant taxa at the family level. Overall, the reported total relative abundance for each study was at least 90% at the phylum taxonomic rank. Consequently, the minor and/or unclassified/not reported/unknown portion of the community was detected as 10% and separately classified as “Other”.

**Figure 4.**
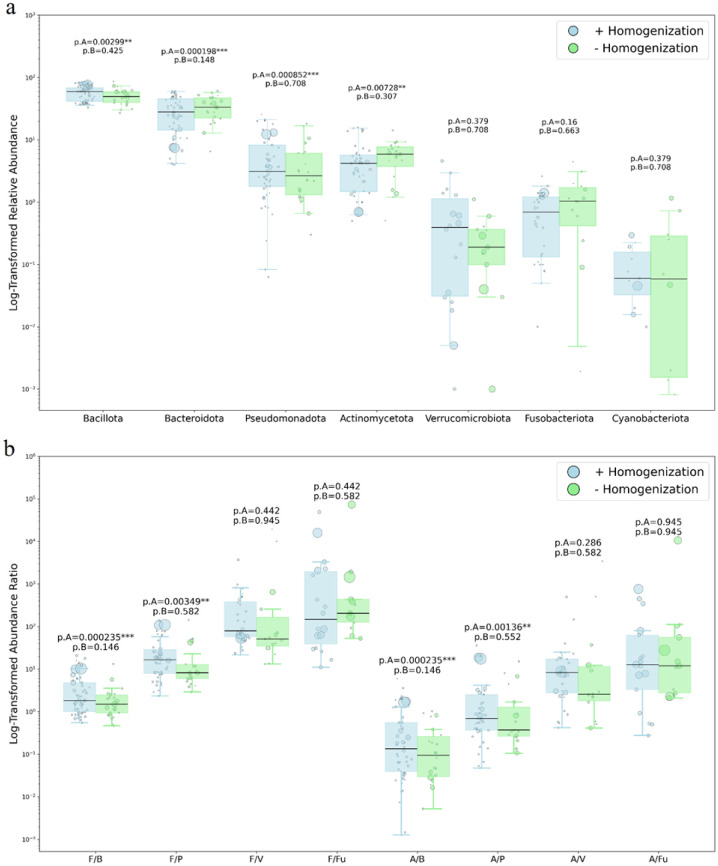
Major phyla relative abundance (a) and Gram-positive to Gram-negative phyla ratio (b) for the data obtained with and without the mechanical homogenization step. P-values shown are FDR-adjusted WLS significance for the complete dataset (p.A) and for the balanced subset excluding two overweighted studies (p.B). F/B: Bacillota [Firmicutes]/Bacteroidota, F/P: Bacillota [Firmicutes]/Pseudomonadota, F/V: Bacillota [Firmicutes]/Verrucomicrobiota, F/Fu: Bacillota [Firmicutes]/Fusobacteriota, A/B: Actinomycetota/Bacteroidota, A/P: Actinomycetota/Pseudomonadota, A/V: Actinomycetota/Verrucomicrobiota, A/Fu: Actinomycetota/Fusobacteriota.

The relative abundances of the predominant bacterial and archaeal phyla were weighted individually based on the sample size of the subjects and separately for the complete dataset and for the balanced subset, excluding exceptionally large studies. The meta-analysis of well-represented phyla and families' relative abundance and their correlation with sample preparation methods is presented in Figure 4a and Tables 1 (sections 1 and 2) and 3 (sections 1, 2, 4, and 5), respectively, along with the Gram-positive to Gram-negative phyla ratios shown in Figure 4b and Table 2. The systematic review of microbiome composition at the major phylum and family levels based on the sample preparation method is displayed in Tables 1 (section 3, phylum level) and 3 (sections 3 and 6, family level).

**Table 1.** Meta-analysis of major phyla relative abundance (with or without mechanical homogenization step).

Phylum	Without homogenization step		With homogenization step	
	Relative abundance	Reference	Relative abundance	Reference
	1. Median (IQR)
Bacillota, %	49.51 (40.08–58.81)	[12],[37]–[41],[43],[45]–[49],[57]–[68]	59.62 (40.56–68.08)	[17],[50],[69]–[114]
Bacteroidota, %	33.4 (22.50–46.93)	[12],[37]–[40],[43],[45]–[49],[57]–[68]	28.03 (14.58–45.42)	[17],[50],[69]–[107],[109]–[114]
Pseudomonadota, %	5.9 (3.75–7.70)	[37]–[41],[43],[45]–[48],[59]–[68]	4.2 (1.44–5.68)	[17],[50],[69]–[81],[83]–[93],[96],[98]–[101],[103]–[107],[109]–[114]
Actinomycetota, %	2.65 (1.31–6.10)	[37]–[40],[43],[45]–[48],[57]–[59],[61]–[68]	3.37 (1.77–8.21)	[17],[50],[69]–[71],[74]–[81],[83]–[91],[93],[94],[96],[97],[99],[101]–[104],[106]–[114]
Verrucomicrobiota, %	1.04 (0.42–1.70)	[37],[39],[43],[45],[59]–[68]	0.545 (0.12–1.20)	[74]–[81],[83],[84],[86]–[91],[93],[94],[99],[103],[106],[107],[109],[110],[112]–[114]
Fusobacteriota [Fusobacteria], %	0.19 (0.10–0.36)	[39],[43],[45],[48],[61]–[68]	0.42 (0.03–1.04)	[74],[78],[81],[96],[99],[105],[106],[108]–[110],[112]–[114]
Mycoplasmatota [Tenericutes], %	0.0245 (0.00–0.05)	[43],[46],[48],[61]–[63],[67]	0.12 (0.04–0.27)	[17],[69],[74],[80],[84],[89],[90],[99],[106],[109],[110],[114]
Cyanobacteriota [Cyanobacteria], %	0.0585 (0.00–0.29)	[39],[43],[45],[48],[61],[63],[64],[66],[67]	0.05745 (0.02–0.14)	[17],[69],[78],[80],[81],[89],[90],[102],[106],[110],[113]
	2. Individual values/Median (IQR)	
Euryarchaeota, %	0.24, 0.043, 0.04, 0.03, 0.00015	[39],[43],[48],[68]	0.08375 (0.04–0.45)	[74],[75],[78],[80],[81],[89]–[91],[106],[113],[114]
	3. Individual values
Crenarchaeota, %	2.00, 0.02, 0.01	[48],[59],[60]	0.00, 0.0005, 0.0011	[114]
Campylobacterota, %	0.01, 0.024	[64],[66]	0.05, 0.00	[78],[113]
Desulfobacterota [Thermodesulfobacteria], %	0.00, 0.00, 0.01, 0.16, 0.26	[39],[43],[64],[66],[68]	1.68, 0.31, 0.10	[78],[91],[113]
Synergistota [Synergistetes], %	0.00, 0.00, 0.01, 0.02	[43],[48],[66]	0.004, 0.005, 0.005, 0.005 0.02, 0.05, 0.10, 0.11	[69],[78],[81],[89],[99],[114]
Lentisphaerota [Lentisphaerae], %	0.00, 0.00, 0.00, 0.0002	[43],[48],[67]	0.00, 0.00, 0.0003, 0.0024, 0.01, 0.02, 0.20	[74],[89],[99],[114]
Nitrospinota [Nitrospinae], %	0.00, 0.00	[43]	0.00, 0.00, 0.0011, 0.16	[89],[114]
Acidobacteriota, %	0.00025, 0.000517	[43]	0.0016, 0.003, 0.0035, 0.0057, 0.05, 0.41	[78],[91],[96],[114]

**Table 2.** Major phyla gram-positive/gram-negative phyla ratios (with or without mechanical homogenization step).

Ratio	Without homogenization step		With homogenization step	
	Median	IQR	Median	IQR
F/B ratio	1.51	(0.93–2.48)	1.82	(1.00–4.73)
F/P ratio	8.23	(5.96–13.03)	17.00	(7.98–29.37)
F/V ratio	51.04	(34.80–164.88)	78.20	(58.79–369.00)
F/Fu ratio	205.21	(125.98–435.00)	88.10	(40.86–1844.89)
A/B ratio	0.05	(0.02–0.20)	0.08	(0.02–0.42)
A/P ratio	0.35	(0.21–0.89)	0.57	(0.17–2.07)
A/V ratio	2.50	(1.13–10.78)	8.28	(2.85–17.12)
A/Fu ratio	11.89	(2.72–55.69)	9.86	(1.52–19.50)

**Table 3.** Relative abundance of significant gram-positive and gram-negative taxa at the family level (with or without mechanical homogenization step).

Family (gram-type)	With homogenization step		Without homogenization step	
	Relative abundance, %	Reference	Relative abundance, %	Reference
	1. Median (IQR)
Lachnospiraceae (+)	16.94 (12.03–27.85)	[17],[69],[71]–[74],[76],[78],[79],[81],[82],[84],[89],[90],[92],[95],[96],[99],[101],[107],[108],[115]–[119]	17.78 (11.91–24.54)	[38]–[40],[57],[59],[64],[67],[120],[121]
Bifidobacteriaceae (+)	1.69 (0.83–2.39)	[17],[74],[76],[78],[79],[81],[84],[89],[90],[96],[101],[107],[108],[119]	2.30 (0.92–3.95)	[39],[40],[58],[64],[67],[120],[121]
Clostridiaceae (+)	1.25 (0.80–2.34)	[17],[74],[78],[81],[82],[84],[89],[96],[101],[116]	0.92 (0.33–1.31)	[37],[39],[40],[59],[64],[67],[120]
	2. Individual values/Median (IQR)	
Erysipelotrichaceae (+)	1.83 (0.59–3.35)	[17],[69],[74],[78],[79],[81],[84],[89],[90],[101],[119]	0.54, 0.78, 0.78, 0.89, 2.89	[59],[64],[67],[120],[121]
Streptococcaceae (+)	1.54 (0.58–1.96)	[17],[78],[81],[84],[89],[90],[107],[119]	0.30, 0.45, 1.85, 1.98	[59],[64],[67],[120]
Peptostreptococcaceae (+)	0.98 (0.64–1.73)	[17],[69],[71],[78],[79],[81],[89],[92],[95],[117]	0.2, 0.3, 0.46, 0.92, 1.63, 7.78	[39],[59],[64],[67],[120],[122]
Coriobacteriaceae (+)	0.92 (0.31–2.67)	[17],[74],[78],[81],[84],[89],[90],[119],[123]	0.35, 0.57, 1.94, 7.17,	[59],[64],[67],[120]
Lactobacillaceae (+)	0.42 (0.24–0.73)	[17],[78],[81],[89],[95],[96],[102],[107],[119]	0.08, 2.81, 0.94	[64],[67],[120]
	3. Individual values
Eubacteriaceae (+)	0.003, 0.006, 3.03, 3.45	[17],[78],[81],[89]	0.005, 0.27, 9.05	[64],[67],[120]
Enterococcaceae (+)	0.0006, 0.01, 0.03, 0.04, 0.05, 0.19	[17],[78],[81],[89],[107],[119]	0.03, 1.08	[64],[120]
Actinomycetaceae (+)	0.00071, 0.02, 0.07	[17],[78],[81]	0.01, 0.06, 0.07, 0.27	[58],[64],[67],[120]
Peptococcaceae (+)	0.02, 0.06, 0.154, 0.27, 0.51	[17],[78],[81],[89],[119]	0.002, 0.01, 0.03	[59],[64],[67]
Methanobacteriaceae	0.05, 0.1, 0.13, 0.55, 1.05	[17],[74],[78],[81]	0.24, 0.31	[39],[120]
	4. Median (IQR)			
Bacteroidaceae (–)	15.25 (11.47–27.19)	[71]–[74],[76],[78],[79],[81],[84],[89],[90],[96],[99],[101],[107],[119]	21.98 (13.04–27.86)	[37]–[40],[57],[64],[67],[120]
Oscillospiraceae/Ruminococcaceae (–)	17.70 (15.19–20.00)	[17],[69],[71]–[74],[76],[78],[79],[81],[82],[84],[89],[90],[92],[95],[96],[98],[99],[101],[107],[108],[115]–[118]	19.42 (14.00–25.60)	[37]–[41],[57],[59],[64],[120]
Prevotellaceae (–)	8.78 (2.80–13.77)	[17],[71],[72],[74],[78],[79],[81],[84],[89],[90]	9.87 (2.72–12.59)	[37],[39],[40],[42],[64],[67],[120]
Rikenellaceae (–)	2.75 (1.79–4.05)	[17],[69],[71],[72],[74],[78],[79],[81],[84],[89],[96],[101],[116],[119]	2.61 (2.01–3.76)	[37],[39],[40],[42],[59],[64],[67],[120],[124]
Veillonellaceae (–)	2.69 (1.20–4.03)	[17],[74],[78],[79],[81],[84],[89],[92],[96],[99],[101],[107]	1.74 (1.06–2.22)	[37],[39],[40],[59],[64],[67],[120]
Enterobacteriaceae (–)	1.14 (0.43–2.43)	[17],[69],[71],[72],[74],[78],[81],[84],[89],[92],[96],[99],[101],[107]	1.86 (1.14–2.63)	[37],[39]–[41],[59],[64],[67],[120],[124],[125]
Christensenellaceae (–)	0.57 (0.42–2.10)	[17],[74],[78],[81],[103],[115],[119]	1.19 (0.51–1.51)	[37],[39],[59],[64],[67],[120],[126]
	5. Individual values/Median (IQR)	
Porphyromonadaceae (–)	0.90 (0.49–1.35)	[17],[74],[78],[79],[81],[84],[89]	0, 0.005, 0.15, 0.97	[59],[64],[67],[120]
Desulfovibrionaceae (–)	0.10 (0.05–0.15)	[17],[74],[78],[81],[84],[89],[119]	0.01, 0.0013, 0.05, 0.17, 0.26	[37],[39],[59],[64],[67]
	6. Individual values
Fusobacteriaceae (–)	0.00, 0.00, 0.03, 0.03, 1.3	[74],[78],[81],[99]	0.0032, 0.03, 0.17, 0.29, 0.36, 1.11	[39],[59],[64],[67],[120],[121]
Akkermansiaceae (–)	0.14, 0.20, 0.96,	[78],[107],[119]	1.11, 1.51, 1.80	[39],[64],[67]
Odoribacteraceae (–)	0.3, 0.0498, 0.44, 0.66	[17],[74],[84]	0.03, 1.6	[40],[67]
Paraprevotellaceae (–)	0.04, 0.26, 0.27, 0.42, 1.6	[17],[74],[81],[84]	0.05	[59]
Verrucomicrobiaceae (–)	0.03, 0.04, 0.1, 0.2, 1.35	[74],[81],[84],[89]		

Complete dataset values with exceptionally large studies (n = 893 and n = 582 from the same research group) at the phylum level showed an expected significant increase in abundance of Gram-positive phyla Bacillota (p.A = 0.003) and decreases in Gram-negative phyla Bacteroidota (p.A = 0.002) upon mechanical homogenization. However, a significant, opposite-to-expected shift was observed, with decreased representation of Actinomycetota (p.A = 0.007) and increased representation of Pseudomonadota (p.A < 0.001) in the presence of the bead-beating step. The corresponding phylum-ratio boxplots (F/B, F/P, F/V, F/Fu, A/B, A/P, A/V, A/Fu) reveal statistically significant shifts attributable to the sample preparation method for the F/B, F/P, A/B, and A/P ratio after correction for multiple testing (p.A < 0.001, p.A = 0.003, p.A < 0.001, p.A = 0.001, respectively). However, this statistical significance results from the substantial imbalance in sample sizes between the groups (see balanced p-values on the Figure 4a). No statistically significant changes in the relative abundance of any bacterial phylum or ratio between the two groups were observed if the balanced subset excluding exceptionally large studies was used (all p > 0.05). A supplementary Mann-Whitney U test was performed as a conservative validation step; it did not identify statistically significant differences (all p.A > 0.05). However, this does not refute the findings from the weighted least squares estimates, given the lower statistical power of the Mann-Whitney test.

Notably, only a limited number of taxa at the family level exhibited any dependence of Gram-type relative abundance on the presence or absence of pre-homogenization. The initial hypothesis found only indirect support: Across the studies considered, the application of bead beating resulted in a greater number of detected taxa for both Gram-positive and Gram-negative groups compared to protocols without mechanical disruption.

### 3.3. Correlations of key representatives of the normal human gut at the phylum and family levels with donor geographic location: Generalized results do not correlate with one-study quantitative findings

**Figure 5.**
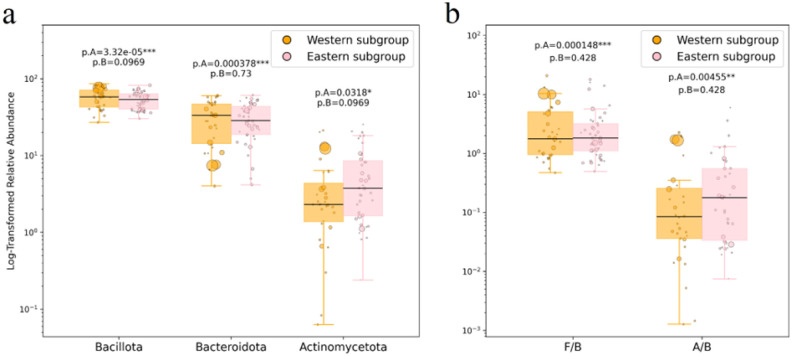
Major phyla relative abundance (a) and Gram-positive to Gram-negative phyla ratio (b) for “Eastern” and “Western” subgroups, collectively with and without the mechanical homogenization step. P-values shown are FDR-adjusted WLS significance for the complete dataset (p.A) and for the balanced subset excluding two overweighted studies (p.B). Only the phyla with 8+ samples in each group are shown (see Methods). F/B: Bacillota [Firmicutes]/Bacteroidota, A/B: Actinomycetota/Bacteroidota.

Meta-analyses of microbiome compositions for Western and Eastern diet groups, based on pooled datasets for phylum-level comparisons (combining both bead beating and non-bead beating protocols), are presented in Figure 5a and Table 4 (sections 1 and 2). The Gram-positive to Gram-negative phyla ratio (meta-analysis) is shown in Figure 5b and Table 5. Family-level comparisons are presented in Table 6 (sections 1, 2, 4, and 5). The systematic review of individual studies examining regional differences is shown in Table 4 (section 3, phylum level) and Table 6 (sections 3 and 6, family level).

**Table 4.** Major Phyla Relative Abundance for the “Western subgroup” and “Eastern subgroup” Donors.

Phylum	“Western subgroup” donors		“Eastern subgroup” donors	
	Relative abundance	Reference	Relative abundance	Reference
	1. Median (IQR)
Bacillota, %	58.05 (42.93–71.03)	[12],[17],[37]–[41],[45],[57],[58],[69]–[84],[86]–[91],[127]	50.9 (39.05–61.65)	[43],[46]–[50],[59]–[68],[92]–[114]
Bacteroidota, %	33.21 (14.29–46.89)	[12],[17],[37]–[40],[45],[57],[58],[69]–[84],[86]–[91],[127]	28.02 (20.76–41.68)	[43],[46]–[50],[59]–[68],[92]–[107],[109]–[114]
Pseudomonadota, %	3.36 (1.25–5.01)	[17],[37]–[41],[45],[69]–[81],[83],[84],[86],[87],[89]–[91],[127]	5.63 (2.47–8.35)	[43],[46]–[48],[50],[59]–[68],[92],[93],[96],[98]–[101],[103]–[107],[109]–[114]
Actinomycetota, %	2.31 (1.33–5.47)	[17],[37]–[40],[45],[57],[58],[69]–[71],[74]–[81],[83],[84],[86]–[91],[127]	3.75 (1.66–8.20)	[43],[46]–[48],[50],[59],[61]–[68],[93],[94],[96]–[99],[101]–[104],[106]–[114]
Verrucomicrobiota, %	1.06 (0.19–1.36)	[37],[39],[45],[74]–[81],[83],[84],[86]–[91]	0.59 (0.12–1.36)	[43],[48],[60]–[68],[93],[94],[99],[103],[106],[107],[109],[110],[112]–[114]
Mycoplasmatota, %	0.27 (0.23–0.39)	[17],[69],[74],[80],[84],[89],[90]	0.03 (0.00–0.07)	[43],[46],[48],[61]–[63],[67],[99],[106],[109],[110],[114]
Cyanobacteriota, %	0.08 (0.05–0.29)	[17],[39],[45],[69],[78],[80],[81],[89],[90]	0.05 (0.00–0.24)	[43],[48],[61],[63],[64],[66],[67],[102],[106],[110],[113]
	2. Individual values/Median (IQR)	
Fusobacteriota, %	0.001, 0.005, 0.025, 0.04, 0.19, 0.29	[39],[45],[74],[78],[81]	0.41 (0.14–1.16)	[43],[48],[61]–[68],[96],[99],[105],[106],[108]–[110],[112]–[114]
Euryarchaeota, %	0.18 (0.04–0.98)	[39],[74],[75],[78],[80],[81],[89]–[91]	0.00, 0.00015, 0.03, 0.04, 0.04, 0.043, 0.06, 0.11, 0.14	[43],[48],[68],[106],[113],[114]
	3. Individual values
Crenarchaeota, %			0.00, 0.0005, 0.0011, 0.01, 0.02, 2.00	[48],[59],[60],[114]
Desulfobacterota, %	0.17, 1.68	[39],[78],[91]	0.00, 0.00, 0.01, 0.10, 0.16, 0.26	[43],[64],[66],[68],[113]
Synergistota, %	0.02, 0.05, 0.004, 0.11	[69],[78],[81],[89]	0.00, 0.00, 0.005, 0.005, 0.005, 0.005, 0.02, 0.10	[43],[48],[66],[99],[114]
Acidobacteriota, %	0.002, 0.41	[78],[91]	0.00025, 0.0005, 0.003, 0.0035, 0.006, 0.05	[43],[96],[114]
Campylobacterota, %	0.05	[78]	0.00, 0.01, 0.02	[64],[66],[113]
Lentisphaerota, %	0.01, 0.02, 0.2	[74],[89]	0.00, 0.00, 0.00, 0.00, 0.00, 0.0002, 0.0003, 0.0024	[43],[48],[67],[99],[114]
Nitrospinota, %	0.16	[89]	0.00, 0.00, 0.00, 0.00, 0.0011	[43],[114]

**Table 5.** Major phyla Gram-positive/Gram-negative phyla ratio for the “Western subgroup” and “Eastern subgroup” donors.

Ratio	Without homogenization step		With homogenization step	
	Median	IQR	Median	IQR
F/B ratio	1.76	(0.95–5.09)	1.82	(1.13–3.14)
F/P ratio	17.00	(8.39–58.21)	9.09	(5.77–22.90)
F/V ratio	62.46	(44.40–252.12)	70.00	(40.33–369.00)
F/Cy ratio	505.23	(238.49–1198.54)	838.57	(193.74–3700.00)
A/B ratio	0.05	(0.01–0.25)	0.10	(0.03–0.44)
A/P ratio	0.55	(0.19–1.64)	0.58	(0.24–1.96)
A/V ratio	2.95	(0.92–8.84)	8.23	(2.50–14.00)
A/Cy ratio	14.55	(8.28–243.37)	113.85	(23.62–500.00)

**Table 6.** Relative abundance of major Gram-positive and Gram-negative taxa at the family level for the “Western subgroup” and “Eastern subgroup” donors.

Family (gram-type)	“Western subgroup” donors		“Eastern subgroup” donors	
	Relative abundance, %	Reference	Relative abundance, %	Reference
	1. Median (IQR)
Lachnospiraceae (+)	15.20 (11.90–23.39)	[17],[38]–[40],[57],[69],[71]–[74],[76],[78],[79],[81],[82],[84],[89],[90],[115]–[117]	24.60 (10.43–30.23)	[59],[64],[67],[92],[95],[96],[99],[101],[107],[108],[118]–[121]
Erysipelotrichaceae (+)	1.74 (0.33–2.55)	[17],[69],[74],[78],[79],[81],[84],[89],[90]	2.89 (2.52–3.95)	[59],[64],[67],[101],[119]–[121]
Bifidobacteriaceae (+)	1.63 (0.70–2.57)	[17],[39],[40],[58],[74],[76],[78],[79],[81],[84],[89],[90]	2.09 (0.85–3.03)	[64],[67],[96],[101],[107],[108],[119]–[121]
Peptostreptococcaceae (+)	0.89 (0.60–1.06)	[17],[39],[69],[71],[78],[79],[81],[89],[117],[122]	1.10 (0.45–3.00)	[59],[64],[67],[92],[95],[120]
	2. Individual values/Median (IQR)	
Clostridiaceae (+)	1.00 (0.80–2.28)	[17],[37],[39],[40],[74],[78],[81],[82],[84],[89],[116]	0.56, 0.63, 0.96, 1.21, 1.9, 2.13	[59],[64],[67],[96],[101],[120]
Coriobacteriaceae (+)	0.62 (0.22–2.73)	[17],[74],[78],[81],[84],[89],[90]	0.01, 0.36, 0.57, 1.95, 2.15, 7.18	[59],[64],[67],[119],[120],[123]
Lactobacillaceae (+)	0.06, 0.23, 0.42, 1.31	[39],[78],[81],[89]	0.65 (0.47–2.63)	[64],[67],[95],[96],[102],[107],[119],[120]
	3. Individual values	
Eubacteriaceae (+)	0.01, 0.01, 3.03, 3.45	[17],[78],[81],[89]	0.0055, 0.27, 9.05	[64],[67],[120]
Streptococcaceae (+)	0.04, 0.30, 0.581, 1.62, 2.29, 6.13	[17],[78],[81],[84],[89],[90]	0.30, 0.45, 0.57, 1.54, 1.85, 1.98	[59],[64],[67],[107],[119],[120]
Enterococcaceae (+)	0.0006, 0.01, 0.05, 0.19	[17],[78],[81],[89]	0.03, 0.03, 0.04, 1.08	[64],[64],[107],[120]
Actinomycetaceae (+)	0.0007, 0.02, 0.06, 0.07	[17],[58],[78],[81]	0.01, 0.07, 0.28	[64],[67],[120]
Peptococcaceae (+)	0.02, 0.06, 0.15, 0.27, 0.51	[17],[78],[81],[89],[119]	0.00195, 0.01, 0.03	[59],[64],[67]
	4. Median (IQR)
Bacteroidaceae (–)	20.29 (12.64–28.48)	[37]–[40],[57],[71]–[74],[76],[78],[79],[81],[84],[89],[90],[119]	12.57 (7.10–22.97)	[64],[67],[96],[99],[101],[107],[120]
Oscillospiracea/Ruminococcaceae (–)	17.30 (15.25–19.79)	[17],[37]–[41],[57],[69],[71]–[74],[76],[78],[79],[81],[82],[84],[89],[90],[115]–[117]	19.42 (8.88–22.15)	[59],[64],[92],[95],[96],[98],[99],[101],[107],[108],[118],[120]
Prevotellaceae (–)	5.89 (2.20–10.72)	[17],[37],[39],[40],[42],[71],[72],[74],[78],[79],[81],[84],[89],[90]	12.65 (4.75–14.69)	[64],[67],[92],[96],[98],[99],[102],[107],[118],[120]
Veillonellaceae (–)	1.67 (0.95–2.50)	[17],[37],[39],[78],[79],[81],[84],[89],[90]	3.30 (2.13–5.95)	[59],[64],[67],[92],[96],[99],[101],[107],[120]
Enterobacteriaceae (–)*	0.88 (0.46–1.37)	[17],[37],[39]–[41],[58],[69],[71],[72],[78],[81],[84],[89],[124],[125]	3.20 (2.41–3.80)	[59],[64],[67],[92],[96],[99],[101],[107],[120]
	5. Individual values/Median (IQR)	
Rikenellaceae (–)	2.90 (2.68–4.24)	[17],[37],[39],[40],[42],[69],[71],[72],[74],[78],[79],[81],[84],[89],[116],[119],[124]	0.26, 0.38, 0.54, 0.9, 0.97, 1.77	[59],[64],[67],[96],[101],[120]
Christensenellaceae (–)	1.25 (0.40–1.92)	[17],[37],[39],[74],[78],[81],[115],[119]	0.008, 0.03, 0.30, 0.5, 1.14, 1.6	[59],[64],[67],[103],[120],[126]
Porphyromonadaceae (–)	0.90 (0.49–1.35)	[17],[74],[78],[79],[81],[84],[89]	0.00, 0.005, 0.15, 0.97	[59],[64],[67],[120]
Desulfovibrionaceae (–)	0.10 (0.02–0.15)	[17],[37],[39],[74],[78],[81],[84],[89],[119]	0.0013, 0.05, 0.26	[59],[64],[67]
	6. Individual values	
Akkermansiaceae (–)	0.20, 0.96, 1.51	[39],[78],[119]	0.14, 1.11, 1.80	[64],[67],[107]
Odoribacteraceae (–)	0.05, 0.30, 0.44, 0.66, 1.60	[17],[40],[74],[84]	0.03	[67]
Paraprevotellaceae (–)	0.04, 0.26, 0.27, 0.42, 1.60	[17],[74],[81],[84]	0.05	[59]
Verrucomicrobiaceae (–)	0.03, 0.04, 0.10, 0.20, 1.35	[74],[81],[84],[89]	-	-
Methanobacteriaceae	0.05, 0.10, 0.13, 0.24, 0.55, 1.05	[17],[39],[74],[78],[81]	0.32	[120]
Fusobacteriaceae (–)	0.00, 0.00, 0.03, 0.03, 0.29	[39],[74],[78],[81]	0.00, 0.03, 0.17, 0.36, 1.11, 1.30	[59],[64],[67],[99],[120],[121]

*statistically significant change (p.A < 0.001).

For the merged and complete datasets comprising “Western subgroup” and “Eastern subgroup” donors, significant relationship between cohort location and relative abundance at the phylum level was detected for Bacillota, Bacteroidota (p.A < 0.001), Actinomycetota (p.A = 0.032), and also for the Gram-positive to Gram-negative ratios (p.A < 0.001 for F/B and p.A = 0.004 for A/B). Consistent with our expectations, no significant changes were observed for these phyla or their ratios in the merged balanced subsets (see balanced p-values in Figure 5a,b).

At the family level, a notable difference in relative abundance was observed for only the Enterobacteriaceae family, which prevailed among the “Eastern subgroup” donors (see Table 6).

**Figure 6.**
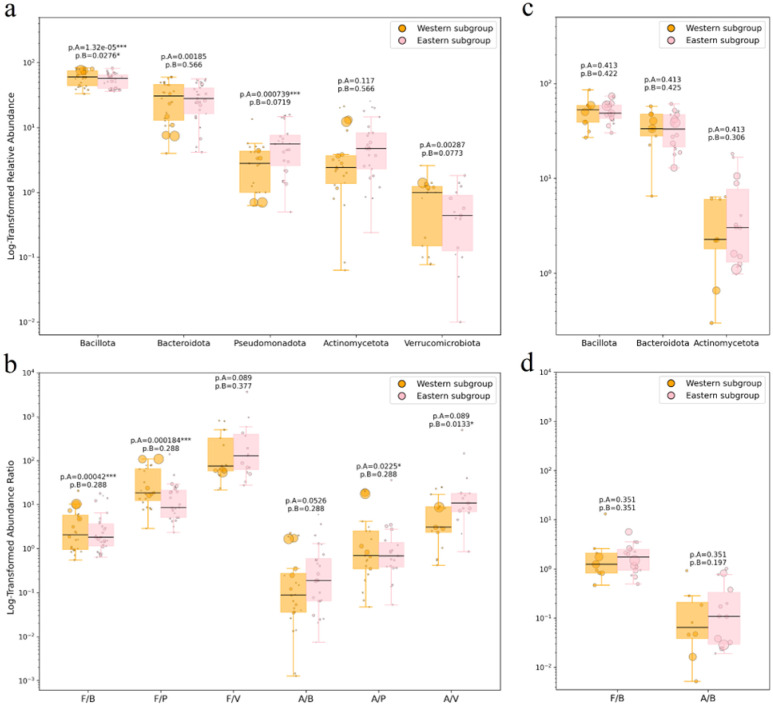
Major phyla relative abundance and Gram-positive to Gram-negative phyla ratio for “Eastern” and “Western” subgroups, with (a, b) and without (c, d) the mechanical homogenization step. P-values shown are FDR-adjusted WLS significance for the complete dataset (p.A) and for the balanced subset excluding two overweighted studies (p.B). Only the phyla with 8+ samples in each group are shown (see Methods). F/B: Bacillota [Firmicutes]/Bacteroidota, F/P: Bacillota [Firmicutes]/Pseudomonadota, F/V: Bacillota [Firmicutes]/Verrucomicrobiota, A/B: Actinomycetota/Bacteroidota, A/P: Actinomycetota/Pseudomonadota, A/V: Actinomycetota/Verrucomicrobiota.

**Figure 7.**
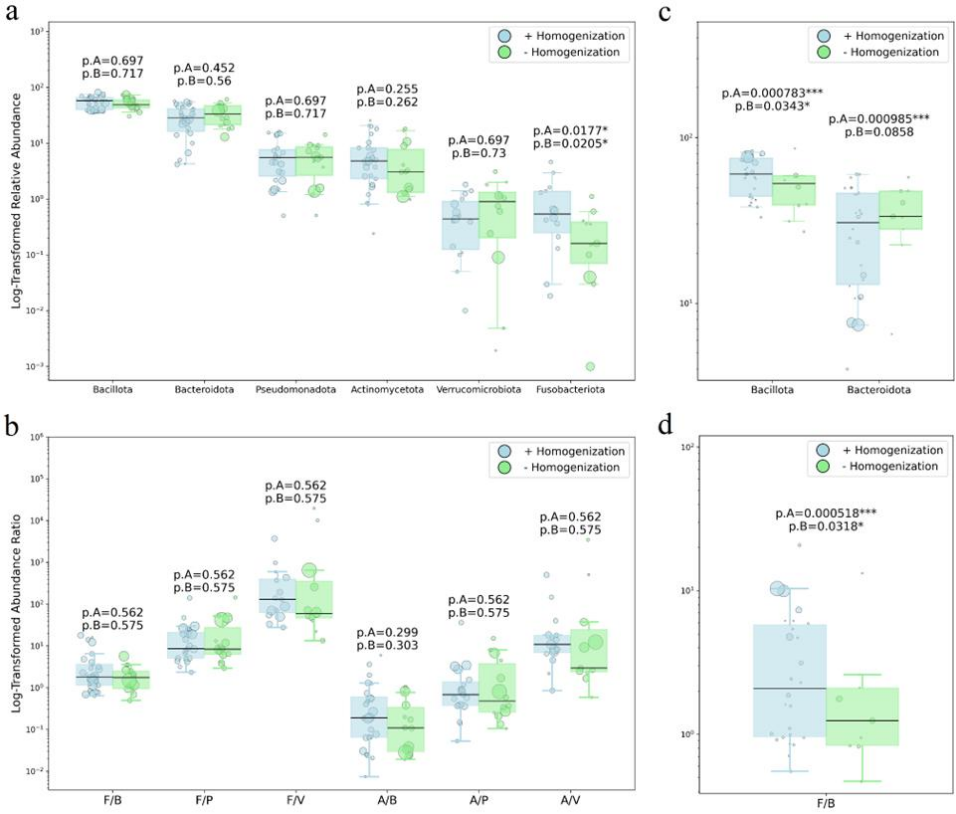
Phyla relative abundance and Gram-positive to Gram-negative phyla ratio within the “Eastern” (a, b) and “Western” (c, d) subgroups, comparing samples with and without mechanical homogenization. P-values shown are FDR-adjusted WLS significance for the complete dataset (p.A) and for the balanced subset excluding two overweighted studies (p.B). Only the phyla with 8+ samples in each group are shown (see Methods). F/B: Bacillota [Firmicutes]/Bacteroidota, F/P: Bacillota [Firmicutes]/Pseudomonadota, F/V: Bacillota [Firmicutes]/Verrucomicrobiota, A/B: Actinomycetota/Bacteroidota, A/P: Actinomycetota/Pseudomonadota, A/V: Actinomycetota/Verrucomicrobiota.

Meta-analysis results for the well-represented phyla and Gram-type ratios across the location-based subgroups are presented in Figure 6a and 6b for samples processed with bead beating, and in Figure 6c and 6d for those processed without this step. Within the “Eastern subgroup”, the comparison of data with and without the mechanical homogenization step is presented in Figure 7a and 7b; the same comparison for the “Western subgroup” is shown in Figure 7c and 7d.

In the complete subset of studies employing bead beating, those representing the “Eastern subgroup” (predominantly plant-based diet) demonstrated statistically significant decrease in the Pseudomonadota phylum compared to the “Western subgroup” (p.A < 0.001). Contrary to our expectations, the “Western subgroup” was associated with an elevated abundance of the phylum Bacillota, exclusively in the presence of the mechanical homogenization step (p.A < 0.001). Nevertheless, none of the differences in relative phyla representation between the two balanced groups reached statistical significance.

The ratio of Gram-positive Bacillota to Gram-negative Bacteroidota, as well as the F/P and A/P ratios, were statistically significant within the complete dataset but not within the balanced one. In contrast, the A/V ratio reached statistical significance only after the exclusion of exceptionally large studies (p.A = 0.089 vs p.B = 0.01, see Figure 6b. No statistically detectable effects were observed in the meta-analysis of studies without the mechanical homogenization step (see Figure 6c and 6d).

In the “Eastern subgroup”, the bead beating step had no significant effect on phylum-level composition or ratios, except for Fusobacteriota which was significantly higher with bead beating in both datasets.

The “Western subgroup” exhibited method-related differences at the well-represented phylum level: Inclusion of the bead beating step led to an increase in the Gram-positive phylum Bacillota (as well as F/B ratio) and a decrease in the Gram-negative phylum Bacteroidota (p.A = 0.001 each, see Figure 7b in the full dataset). However, these associations were largely driven by two exceptionally large studies, both included in the Western cohort; when a balanced subset excluding these studies was analyzed, the significance for the phylum Bacteroidota was no longer observed.

No other phyla or their ratios showed statistically significant differences within the “Eastern” or “Western” subgroups, in the absence or presence of the “bead beating” stage for balanced subsets.

## 4. Discussion

### 4.1. Meta-analysis outcomes and interpretation of findings

The fecal microbiota is seen as a functional part of the body, and its imbalance is thought to be one of the markers of various pathological processes, with dysbiosis increasingly viewed as a dysfunction of this unified “organ”. It is important to note that even commensal members of the normal gut microbiota may contribute to pathological processes under specific host or environmental conditions. Evidence, including the “driver-passenger” model, shows that dysbiosis within otherwise non-pathogenic communities can promote inflammation and barrier dysfunction and has been linked to autoimmune disorders and colorectal carcinogenesis [91],[108],[128]. Establishing a reliable reference interval for the normal (healthy) gut microbiome, encompassing its microbial composition and the relationships between taxonomic groups, is of paramount importance.

According to the results of the meta-analysis, the microbiota composition at the phylum level is predominantly represented by Gram-positive Bacillota (median 49.5–59.6%, depending on the sampling location and the presence or absence of a pre-homogenization step) and Gram-negative Bacteroidota (28.0–33.4%). These two phyla consistently contribute the largest proportions across all included datasets, irrespective of biosample preparation protocols or geographical origin of participants (Tables 1 and 4). Bacteria from the phyla Pseudomonadota (3.4–5.9%), Actinomycetota (2.3–3.7%), Verrucomicrobiota (0.5–1.0%), Fusobacteriota (up to 4.6%), Euryarchaeota (up to 2.1%) Crenarchaeota (up to 2.0%), Mycoplasmatota (0.02–0.3%), Cyanobacteriota (0.05–0.08%), Campylobacterota (up to 0.05%), Desulfobacterota (up to 1.68%), Synergistota (up to 0.1%), Lentisphaerota (up to 0.2%), Nitrospinota (up to 0.16%), and Acidobacteriota (up to 0.41%), are significantly less abundant.

**Figure 8.**
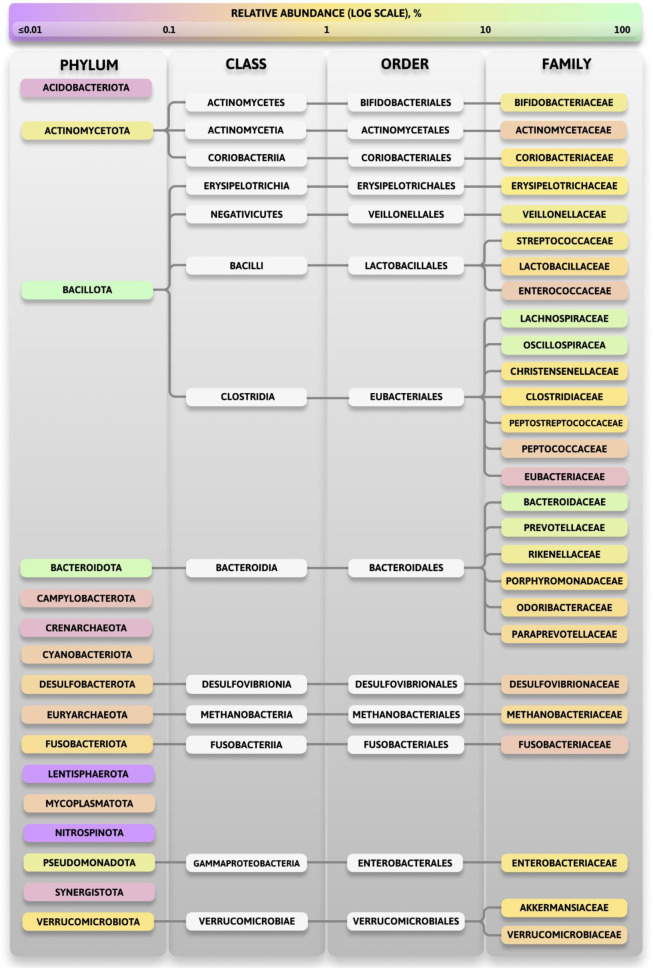
Taxonomy classification chart of key representatives.

Based on the selected reliable studies, we can conclude that the key representatives at the family taxonomic rank are Lachnospiraceae (the dominant family of the Bacillota phylum), Bacteroidaceae (the dominant family of the Bacteroidota phylum), Oscillospiraceae, Prevotellaceae, Bifidobacteriaceae, Coriobacteriaceae, Clostridiaceae, Rikenellaceae, Peptostreptococcaceae, Veillonellaceae, Erysipelotrichaceae, Streptococcaceae, Enterobacteriaceae, Lactobacillaceae, Christensenellaceae Akkermansiaceae, Porphyromonadaceae, Enterococcaceae, Odoribacteraceae and Paraprevotellaceae. Additionally, representatives of the families Fusobacteriaceae, Methanobacteriaceae, Verrucomicrobiaceae, Desulfovibrionaceae, Actinomycetaceae, Peptococcaceae, and Eubacteriaceae are often found in human feces but are less abundant. The key representatives and their updated taxonomy are presented in Figure 8.

Numerous individual population-based studies show correlations between the composition of normal “healthy microbiota” and geographical location (thus specifically referring to diet) when exploring samples from various subjects (study design: “case-control”) or different ethnic groups. Based on other studies, we anticipated a positive association between Bacteroidota and the “Western diet” and a negative correlation with the “Eastern diet”, as well as an inverse trend for Bacillota [7],[10],[15],[18].

However, our synthesis and comparison of multiple studies either did not reveal significant differences in microbiome taxonomic profiles associated with cohort location or demonstrate trends opposite to those predicted by theoretical considerations and prior evidence. One possible explanation for the lack of associations in such analyses is the substantial variation in sample sizes across the included studies, which may obscure genuine patterns and introduce bias. In our case, two particularly large studies (n = 893 and n = 582), both conducted by the same research group, had a disproportionately strong influence on the overall results. To mitigate this effect, we performed separate analyses using a complete dataset and a balanced subset. The latter excluded the oversized cohorts to ensure more comparable contributions of individual studies in the weighted least squares estimates and to reduce the impact of sample size heterogeneity on meta-analytic outcomes.

The “Western subgroup” was associated with an increased abundance of Bacteroidota in several sample variants. However, this association was observed only in datasets dominated by the aforementioned studies from a single research group and not in the balanced dataset, as well as an increase in Verrucomicrobiota and a decrease in Pseudomonadota. Unexpectedly, the “Western subgroup” was also associated with higher levels of Bacillota (as well as higher F/B ratio) exclusively in the presence of the mechanical homogenization step (p.A < 0.001) in all dataset variants. This finding does not align with prior assumptions of a negative correlation between the low-fiber diet (expected in the “Western” group) and the phylum Bacillota [15]. No significant changes were observed for the other phyla in the balanced subsets. Among the phylum-level ratios, only the increase in the Actinomycetota-to-Verrucomicrobiota (A/V) ratio remained statistically significant when comparing the “Eastern subgroup” to the “Western subgroup”.

Notably, articles reporting data on the phyla Euryarchaeota, Desulfobacterota, Lentisphaerota, and Acidobacteriota in studies involving the “Western subgroup” (both with and without the bead-beating step) were scarce compared to those from the “Eastern subgroup.” Similarly, within the “Western subgroup” lacking homogenization, Pseudomonadota, Verrucomicrobiota, and other low-abundance taxa were underrepresented, thereby limiting the feasibility of conducting a robust meta-analysis for these groups. These findings suggest that these phyla either constitute a minor fraction of the intestinal microbiota in populations from European and American regions compared to the “Eastern subgroup,” or remain insufficiently studied in those contexts.

The abovementioned inconsistencies point to deeper methodological challenges in microbiome research. While variations in the choice and number of subjects, as well as limitations in participant selection, undoubtedly influence the assessment of taxonomic representation, technical factors may be equally, if not more, decisive. The generation of reliable and reproducible data in this field is inherently complex, as outcomes are shaped not only by biological variation but also by technical factors across multiple stages of the NGS workflow, including sample collection, storage, DNA extraction, and sequencing protocols.

The introduction of molecular biological techniques has greatly advanced research on the human intestinal microbiome, which has gained exponentially in popularity. However, the variety of methods used and the lack of strict interlaboratory standards for DNA extraction and analysis reduce the likelihood of a reliable meta-analysis of this complex microbial community and its dynamics. These methodological inconsistencies are particularly important at the stage of sample handling and DNA isolation, which play a critical role in shaping the observed taxonomic profiles.

The yield of extracted DNA from complex materials such as feces directly depends on the method and quality of its isolation, as well as on storage conditions and sample preparation. Long-term storage of samples without freezing can lead to the growth of facultative anaerobes and a decrease in the number of strict anaerobes, which may negatively impact the reliability of community analysis results [129].

Extraction methods with inefficient lysis steps significantly affect the detection of Gram-positive bacteria and archaea, the latter sharing structural similarities in cell wall composition with Gram-positive bacteria. Individual studies demonstrate the positive influence of the mechanical homogenization stage on the extraction of genetic material from feces, especially for Gram-positive bacteria DNA yield (which can increase up to 10-fold) [31]–[33].

Surprisingly, however, when the data from different studies were pooled for this meta-analysis, the presence of the mechanical homogenization step was associated only with moderate shifts in the relative abundance of microorganisms at the phylum and family levels, which were likewise dependent on dataset balancing. The generalized results revealed a significant relationship between qualitative and quantitative aspects of microbial composition and the presence of the homogenization step for several phyla only when the complete dataset was considered. Specifically, this applied to elevated Bacillota & decreased Bacteroidota (for merged datasets where samples from both dietary subgroups were pooled, and separately within the subgroup with a Westernised diet), and counterintuitively elevated Gram-negative Pseudomonadota and decreased Gram-positive Actinomycetota (for merged datasets). None of these observations regarding phyla remained significant upon exclusion of the outliers to form the balanced dataset with the exception of the Bacillota phylum; and only in the Western subgroup. The only other significant difference observed in the balanced dataset was an unexpected increase in Fusobacteriota abundance within the “Eastern” group following the application of the bead-beating step.

It was initially anticipated that the presence of the homogenization step (recognized as one of the most effective approaches for cell lysis) would show a significant association with the outcomes of the meta-analysis of the gut microbiota composition. Despite these expectations, the inclusion or omission of the bead-beating did not reveal any consistent differences in taxonomic profiles. These findings suggest that the contribution of this step alone may not be the primary driver of variation observed in individual studies and may be limited by the influence of other stages of the microbiome 16S rRNA gene sequencing workflow.

Other drawbacks associated with the qualitative and quantitative analyses using the 16S rRNA gene sequencing methodology may explain these results [27],[130]–[133]:

1) Universal primers commonly used for amplifying variable regions of the 16S rRNA gene do not anneal equally well across taxa, resulting in uneven amplification efficiency and distortion in microbiota structure results.

2) Additionally, major taxa gain a significant advantage over minor (up to 0.5%) taxa during the initial amplification cycles, which is crucial for the overall process. Consequently, minor taxa may be underrepresented or completely absent. Unfortunately, since each sample has a unique taxa composition, a random error, rather than a systematic error, arises from the aforementioned issues, which cannot be predicted or accounted for in each case.

3) The individual number of 16S rRNA gene copies inherent to each microorganism is rarely taken into consideration, leading to ambiguous estimates of the relative content of taxa at the genus level and above [134].

4) Moreover, the estimation of specific taxa content can vary significantly depending on the database used (for example, RDP or SILVA). For instance, in the RDP database, there is no genus named *Subdoligranulum*, which is classified under the *Oscillospiraceae* family in the SILVA database. Instead, the corresponding reads in the RDP database are classified as the genus *Gemmiger* of the *Ruminococcaceae* family. This heterogeneity is attributed to differences in the nature of database maintenance (manual or automatic) and the entry of sequences (with or without prior confirmation via culture methods).

5) Since the relative content of taxa, rather than absolute content, is estimated, an increase in one taxon automatically leads to an underestimation of the content of others.

6) Finally, the universal primer sequences complementary to the 16S rRNA gene are selected based on already known taxa, which may result in insufficient affinity and sequence specificity of DNA binding for currently unknown representatives of the microbiota.

7) Different regions of the 16S rRNA gene employed for analysis exhibit unequal phylogenetic fidelity.

8) Other modifications of sequencing-based analysis include various options for library preparation, different sequencing techniques (e.g., Platform 454 and Junior “Roche,” SOLiD and Ion Torrent “Applied Biosystems,” HiSeq and MiSeq “Illumina,” among others), and the subsequent assembly of “raw” reads. These factors can lead to differences in data normalization, taxa overestimation, or underrepresentation, particularly at lower taxonomic ranks.

Thus, it is challenging to reliably assess the true contribution of each representative to the biodiversity of the microbial community when systematizing data obtained using different protocols. Moreover, this challenge persists even when utilizing the same protocol (refer to reasons 1, 2, and 5) in fecal microbiome analyses. As we have demonstrated in our paper, we were unable to statistically confirm even the well-established fact that microbiome composition depends on the biosample preparation method when analyzing generalized data obtained through the 16S NGS sequencing procedure.

### 4.2. Study limitations

This study has several limitations that should be considered when interpreting the findings.

First, when analyzing the complete dataset, the results of the meta-analysis were affected by two exceptionally large studies, which significantly exceeded the sample sizes of the remaining included datasets. Although we addressed this effect by analyzing both the full and balanced subsets, large discrepancies in sample size limit the ability to draw generalizable conclusions across all studies, as aggregated results may reflect weighting artifacts rather than consistent biological patterns.

Second, certain low-abundance phyla (e.g., Crenarchaeota, Desulfobacterota, Campylobacterota, Lentisphaerota) were underrepresented in datasets originating from “Western” cohorts, regardless of sample preparation protocol. This constrained the feasibility of comparative analysis for these taxa.

Moreover, although cohort sizes were balanced between plant-based and protein-rich dietary groups to ensure statistical comparability, the underlying studies were unevenly distributed across global regions. While the dataset covered a broad range of countries, some regions, most notably Africa, South America, and Oceania, remain underrepresented due to a lack of studies meeting our inclusion criteria. This reflects a persistent data gap in the field and limits the generalizability of diet-microbiota associations.

Third, the analysis was restricted to the phylum and family taxonomic ranks, as representatives at these levels are the most reliably detected by 16S rRNA sequencing, while less abundant groups (up to 0.5%) may remain undetected due to previously discussed methodological limitations such as primer bias and uneven amplification efficiency.

In addition, despite the use of stringent inclusion and exclusion criteria addressing participant selection and sample handling, substantial variation remained in the technical aspects of the included studies. The reviewed studies differed in several technical parameters, including the targeted 16S rRNA variable regions, sequencing platforms, and DNA extraction protocols. These factors may serve as uncontrolled sources of variation and introduce inconsistency into taxonomic profiling outcomes.

Another important limitation is the absence of standardized dietary metadata in the included studies, we were unable to assess direct correlations between microbiome composition and nutritional intake. As a result, geographic classification (e.g., “Eastern” vs. “Western” cohorts) was used as an indirect indicator of dominant dietary patterns.

Finally, the reported microbial composition was based on relative abundance values derived from sequencing data. The reliance on taxonomic profiling without absolute quantification (such as the actual number of DNA copies per unit of sample) leads to ratio-based distortion of compositional structure, whereby changes in the abundance of one taxon directly affect the measured proportions of others.

## 5. Conclusions

The findings of this meta-analysis demonstrate that, under current conditions, it is not possible to establish reliable reference intervals for the normal gut microbiota by generalizing results from the majority of published studies. This limitation is primarily due to substantial methodological heterogeneity, including differences in sample preparation, DNA extraction, sequencing protocols, and data reporting practices, which obscures true biological patterns and prevents meaningful cross-study comparability.

It is important to emphasize that the failure to confirm expected associations in the generalized dataset does not undermine the validity of the underlying hypotheses. Rather, it highlights the methodological inconsistencies across studies.

Consequently, transitioning from single-study quantitative findings to broader conclusions based on generalizations from multiple studies employing 16S rRNA sequencing may only be reliable if strict control is maintained over all experimental technique parameters across the studies considered. In this context, we deem it necessary to offer two suggestions for improving the reliability and consistency of research results.

The first recommendation is to achieve maximum standardization of the sample preparation methodology and subsequent data processing, adhering to international guidelines. Specifically, when utilizing alternative extraction techniques, it is essential to demonstrate that the resulting microbiome profile resembles that obtained with internationally recommended kits or isolation methods. In particular, mechanical homogenization (“bead beating”) should be universally applied in all microbiome studies.

For the second recommendation, we advocate for the use of precise reference quantitative methods (such as quantitative PCR or droplet digital PCR [135]) for quantitatively assessing taxa representation. When complemented by an absolute quantification method and conducted in a controlled manner, 16S NGS will not only serve as a research tool but also become a globally reproducible methodology for assessing microbial community composition.

Only after accumulating a sufficient number of studies with these considerations addressed can we obtain true reference intervals for normal fecal microbiota. This will enable clinicians to make informed medical judgments regarding fecal microbiota imbalances, except for strictly pathogenic bacteria, where current knowledge is adequate.

## Use of AI tools declaration

The authors declare they have not used Artificial Intelligence (AI) tools in the creation of this article.



## References

[1] The Human Microbiome Project Consortium (2012). A framework for human microbiome research. Nature.

[2] Wang WL, Xu SY, Ren ZG (2015). Application of metagenomics in the human gut microbiome. World J Gastroenterol.

[3] Qin J, Li R, Raes J (2010). A human gut microbial gene catalogue established by metagenomic sequencing. Nature.

[4] McDonald D, Hyde E, Debelius JW (2018). American gut: An open platform for citizen science microbiome research. mSystems.

[5] Tigchelaar EF, Zhernakova A, Dekens JAM (2015). Cohort profile: LifeLines DEEP, a prospective, general population cohort study in the northern Netherlands: Study design and baseline characteristics. BMJ Open.

[6] Shah RM, McKenzie EJ, Rosin MT (2020). An integrated multi-disciplinary perspective for addressing challenges of the human gut microbiome. Metabolites.

[7] Nikonova EL, Popova EN (2019). Microbiota: Monograph.

[8] Yudina YuV, Korsunsky AA, Aminova AI (2019). Gut microbiota as a separate body system. Dokazatelnaya Gastroenterol.

[9] Arumugam M, Raes J, Pelletier E (2011). Enterotypes of the human gut microbiome. Nature.

[10] Rinninella E, Raoul P, Cintoni M (2019). What is the healthy gut microbiota composition? A changing ecosystem across age, environment, diet, and diseases. Microorganisms.

[11] Abenavoli L, Scarpellini E, Colica C (2019). Gut microbiota and obesity: A role for probiotics. Nutrients.

[12] Gallè F, Valeriani F, Cattaruzza MS (2020). Mediterranean diet, physical activity and gut microbiome composition: A cross-sectional study among healthy young Italian adults. Nutrients.

[13] Lozupone CA, Stombaugh JI, Gordon JI (2012). Diversity, stability and resilience of the human gut microbiota. Nature.

[14] Mariat D, Firmesse O, Levenez F (2009). The Firmicutes/Bacteroidetes ratio of the human microbiota changes with age. BMC Microbiol.

[15] Vacca M, Celano G, Calabrese FM (2020). The controversial role of human gut Lachnospiraceae. Microorganisms.

[16] Gill SR, Pop M, Deboy RT (2006). Metagenomic analysis of the human distal gut microbiome. Science.

[17] Imhann F, Vich Vila A, Bonder MJ (2018). Interplay of host genetics and gut microbiota underlying the onset and clinical presentation of inflammatory bowel disease. Gut.

[18] Sheybak VM (2015). Human gut microbes and its influence on metabolism. Educ Establ Grodno State Med Univ.

[19] Feng Q, Liang S, Jia H (2015). Gut microbiome development along the colorectal adenoma–carcinoma sequence. Nat Commun.

[20] Lazar V, Ditu L-M, Pircalabioru GG (2018). Aspects of gut microbiota and immune system interactions in infectious diseases, immunopathology, and cancer. Front Immunol.

[21] Song M, Chan AT, Sun J (2020). Influence of the gut microbiome, diet, and environment on risk of colorectal cancer. Gastroenterology.

[22] Sorbara MT, Littmann ER, Fontana E (2020). Functional and genomic variation between human-derived isolates of Lachnospiraceae reveals inter- and intra-species diversity. Cell Host Microbe.

[23] Elhag DA, Kumar M, Al Khodor S (2020). Exploring the triple interaction between the host genome, the epigenome, and the gut microbiome in type 1 diabetes. Int J Mol Sci.

[24] Mitsou EK, Detopoulou M, Kakali A (2019). Mining possible associations of faecal *A. muciniphila* colonisation patterns with host adiposity and cardiometabolic markers in an adult population. Benef Microbes.

[25] Ottman N, Reunanen J, Meijerink M (2017). Pili-like proteins of *Akkermansia muciniphila* modulate host immune responses and gut barrier function. PLOS ONE.

[26] Topping DL, Clifton PM (2001). Short-chain fatty acids and human colonic function: Roles of resistant starch and nonstarch polysaccharides. Physiol Rev.

[27] Deering KE, Devine A, O'Sullivan TA (2019). Characterizing the composition of the pediatric gut microbiome: A systematic review. Nutrients.

[28] Hiippala K, Jouhten H, Ronkainen A (2018). The potential of gut commensals in reinforcing intestinal barrier function and alleviating inflammation. Nutrients.

[29] Könönen E, Wade WG (2015). Actinomyces and related organisms in human infections. Clin Microbiol Rev.

[30] Stojanov S, Berlec A, Štrukelj B (2020). The influence of probiotics on the Firmicutes/Bacteroidetes ratio in the treatment of obesity and inflammatory bowel disease. Microorganisms.

[31] Bikaeva IO, Zlobovskaya OA, Shipulin GA (2021). The choice of method for DNA isolation from fecal material affects the defined bacterial community composition.

[32] Costea PI, Zeller G, Sunagawa S (2017). Towards standards for human fecal sample processing in metagenomic studies. Nat Biotechnol.

[33] Santiago A, Panda S, Mengels G (2014). Processing faecal samples: A step forward for standards in microbial community analysis. BMC Microbiol.

[34] IHMS (International Human Microbiome Standards).

[35] Dore J, Ehrlich SD, Levenez F (2015). IHMS_SOP 07 V2: Standard operating procedure for fecal samples DNA extraction, Protocol H.

[36] Dore J, Ehrlich SD, Levenez F (2020). IHMS_SOP 06 V3: Standard operating procedure for fecal samples DNA extraction, Protocol Q.

[37] Zakerska-Banaszak O, Tomczak H, Gabryel M (2021). Dysbiosis of gut microbiota in Polish patients with ulcerative colitis: A pilot study. Sci Rep.

[38] Barengolts E, Green SJ, Eisenberg Y (2018). Gut microbiota varies by opioid use, circulating leptin and oxytocin in African American men with diabetes and high burden of chronic disease. PLoS ONE.

[39] Sisti D, Pazienza V, Piccini F (2022). A proposal for the reference intervals of the Italian microbiota “scaffold” in healthy adults. Sci Rep.

[40] Cano-Ortiz A, Laborda-Illanes A, Plaza-Andrades I (2020). Connection between the gut microbiome, systemic inflammation, gut permeability and FOXP3 expression in patients with primary Sjögren's syndrome. Int J Mol Sci.

[41] Borgo F, Riva A, Benetti A (2017). Microbiota in anorexia nervosa: The triangle between bacterial species, metabolites and psychological tests. PLOS ONE.

[42] Hiel S, Bindels LB, Pachikian BD (2019). Effects of a diet based on inulin-rich vegetables on gut health and nutritional behavior in healthy humans. Am J Clin Nutr.

[43] Sugurmar ANK, Mohd R, Shah SA (2021). Gut microbiota in immunoglobulin A nephropathy: A Malaysian perspective. BMC Nephrol.

[44] Pallav K, Dowd SE, Villafuerte J (2014). Effects of polysaccharopeptide from *Trametes versicolor* and amoxicillin on the gut microbiome of healthy volunteers. Gut Microbes.

[45] Santoru ML, Piras C, Murgia A (2017). Cross sectional evaluation of the gut-microbiome metabolome axis in an Italian cohort of IBD patients. Sci Rep.

[46] Hou Q, Zhao F, Liu W (2020). Probiotic-directed modulation of gut microbiota is basal microbiome dependent. Gut Microbes.

[47] Kim SW, Suda W, Kim S (2013). Robustness of gut microbiota of healthy adults in response to probiotic intervention revealed by high-throughput pyrosequencing. DNA Res.

[48] Das T, Jayasudha R, Chakravarthy S (2021). Alterations in the gut bacterial microbiome in people with type 2 diabetes mellitus and diabetic retinopathy. Sci Rep.

[49] Wong VWS, Tse CH, Lam TTY (2013). Molecular characterization of the fecal microbiota in patients with nonalcoholic steatohepatitis—a longitudinal study. PLoS ONE.

[50] Sun XZ, Zhao DY, Zhou YC (2020). Alteration of fecal tryptophan metabolism correlates with shifted microbiota and may be involved in pathogenesis of colorectal cancer. World J Gastroenterol.

[51] Page MJ, McKenzie JE, Bossuyt PM (2021). The PRISMA 2020 statement: An updated guideline for reporting systematic reviews. BMJ.

[52] NCBI [Internet].

[53] Rebrova O. Yu, Fediaeva V. K. (2016). The questionnaire to assess the risk of systematic bias in non-randomized comparative studies: The Russian-language version of the Newcastle-Ottawa scale. Med Technol Assess Choice.

[54] Rebrova OY, Fediaeva VK, Khachatryan GR (2015). Adaptation and validation of the cochrane questionnarie to assess risks of bias in randomized controlled trials. Med Technol Assess Choice.

[55] Shea BJ, Grimshaw JM, Wells GA (2007). Development of AMSTAR: A measurement tool to assess the methodological quality of systematic reviews. BMC Med Res Methodol.

[56] SCImago Journal & Country Rank SCImago, (n.d.). SJR. [Internet].

[57] Senina A, Markelova M, Khusnutdinova D (2024). Two-year study on the intra-individual dynamics of gut microbiota and short-chain fatty acids profiles in healthy adults. Microorganisms.

[58] Reimer RA, Soto-Vaca A, Nicolucci AC (2020). Effect of chicory inulin-type fructan–containing snack bars on the human gut microbiota in low dietary fiber consumers in a randomized crossover trial. Am J Clin Nutr.

[59] Kalyana Chakravarthy S, Jayasudha R, Ranjith K (2018). Alterations in the gut bacterial microbiome in fungal keratitis patients. PLOS ONE.

[60] Li F, Wang M, Wang J (2019). Alterations to the gut microbiota and their correlation with inflammatory factors in chronic kidney disease. Front Cell Infect Microbiol.

[61] Yang Y, Misra BB, Liang L (2019). Integrated microbiome and metabolome analysis reveals a novel interplay between commensal bacteria and metabolites in colorectal cancer. Theranostics.

[62] Liu W, Zhang R, Shu R (2020). Study of the relationship between microbiome and colorectal cancer susceptibility using 16SrRNA sequencing. BioMed Res Int.

[63] Ren Z, Fan Y, Li A (2020). Alterations of the human gut microbiome in chronic kidney disease. Adv Sci.

[64] Jiao B, Cao X, Zhang C (2023). Alterations of the gut microbiota in patients with postherpetic neuralgia. AMB Express.

[65] Yoon H, Park S, Jun YK (2023). Evaluation of bacterial and fungal biomarkers for differentiation and prognosis of patients with inflammatory bowel disease. Microorganisms.

[66] Kumar S, Mahajan S, Kale D (2024). Insights into the gut microbiome of vitiligo patients from India. BMC Microbiol.

[67] Lee SM, Kim N, Yoon H (2021). Compositional and functional changes in the gut microbiota in irritable bowel syndrome patients. Gut Liver.

[68] Yu X, Ge P, Zhai Y (2023). Gut microbiota in adults with Moyamoya disease: Characteristics and biomarker identification. Front Cell Infect Microbiol.

[69] Chávez-Carbajal A, Nirmalkar K, Pérez-Lizaur A (2019). Gut microbiota and predicted metabolic pathways in a sample of Mexican women affected by obesity and obesity plus metabolic syndrome. Int J Mol Sci.

[70] Chávez-Carbajal A, Pizano-Zárate ML, Hernández-Quiroz F (2020). Characterization of the gut microbiota of individuals at different T2D stages reveals a complex relationship with the host. Microorganisms.

[71] Higuchi BS, Rodrigues N, Gonzaga MI (2018). Intestinal dysbiosis in autoimmune diabetes is correlated with poor glycemic control and increased interleukin-6: A pilot study. Front Immunol.

[72] Leite AZ, Rodrigues N de C, Gonzaga MI (2017). Detection of increased plasma interleukin-6 levels and prevalence of *Prevotella copri* and *Bacteroides vulgatus* in the feces of type 2 diabetes patients. Front Immunol.

[73] Grosicki GJ, Riemann BL, Flatt AA (2020). Self-reported sleep quality is associated with gut microbiome composition in young, healthy individuals: A pilot study. Sleep Med.

[74] Bressa C, Bailén-Andrino M, Pérez-Santiago J (2017). Differences in gut microbiota profile between women with active lifestyle and sedentary women. PLOS ONE.

[75] Zhernakova A, Kurilshikov A, Bonder MJ (2016). Population-based metagenomics analysis reveals markers for gut microbiome composition and diversity. Science.

[76] Davis LMG, Martínez I, Walter J (2011). Barcoded pyrosequencing reveals that consumption of galactooligosaccharides results in a highly specific bifidogenic response in humans. PLoS ONE.

[77] Tap J, Mondot S, Levenez F (2009). Towards the human intestinal microbiota phylogenetic core. Environ Microbiol.

[78] Bombin A, Yan S, Bombin S (2022). Obesity influences composition of salivary and fecal microbiota and impacts the interactions between bacterial taxa. Physiol Rep.

[79] Martínez I, Kim J, Duffy PR (2010). Resistant starches types 2 and 4 have differential effects on the composition of the fecal microbiota in human subjects. PLoS ONE.

[80] Bezek K, Petelin A, Pražnikar J (2020). Obesity measures and dietary parameters as predictors of gut microbiota phyla in healthy individuals. Nutrients.

[81] Volokh O, Klimenko N, Berezhnaya Y (2019). Human gut microbiome response induced by fermented dairy product intake in healthy volunteers. Nutrients.

[82] González-Zancada N, Redondo-Useros N, Díaz LE (2020). Association of moderate beer consumption with the gut microbiota and SCFA of healthy adults. Molecules.

[83] Larsen N, Vogensen FK, van den Berg FWJ (2010). Gut microbiota in human adults with type 2 diabetes differs from non-diabetic adults. PLoS ONE.

[84] Hansen TH, Thomassen MT, Madsen ML (2018). The effect of drinking water pH on the human gut microbiota and glucose regulation: Results of a randomized controlled cross-over intervention. Sci Rep.

[85] Granado-Serrano AB, Martín-Garí M, Sánchez V (2019). Faecal bacterial and short-chain fatty acids signature in hypercholesterolemia. Sci Rep.

[86] Bjørkhaug ST, Aanes H, Neupane SP (2019). Characterization of gut microbiota composition and functions in patients with chronic alcohol overconsumption. Gut Microbes.

[87] Dei-Cas I, Giliberto F, Luce L (2020). Metagenomic analysis of gut microbiota in non-treated plaque psoriasis patients stratified by disease severity: Development of a new Psoriasis-Microbiome Index. Sci Rep.

[88] Wasser CI, Mercieca EC, Kong G (2020). Gut dysbiosis in Huntington's disease: Associations among gut microbiota, cognitive performance and clinical outcomes. Brain Commun.

[89] Fernandez-Sanjurjo M, Fernandez J, Martinez-Camblor P (2024). Dynamics of gut microbiota and short-chain fatty acids during a cycling grand tour are related to exercise performance and modulated by dietary intake. Nutrients.

[90] Benedict C, Vogel H, Jonas W (2016). Gut microbiota and glucometabolic alterations in response to recurrent partial sleep deprivation in normal-weight young individuals. Mol Metab.

[91] Ruiz-Saavedra S, Arboleya S, Nogacka AM (2023). Commensal fecal microbiota profiles associated with initial stages of intestinal mucosa damage: A pilot study. Cancers.

[92] Su J, Wang Y, Zhang X (2021). Remodeling of the gut microbiome during Ramadan-associated intermittent fasting. Am J Clin Nutr.

[93] Kurahashi A, Enomoto T, Oguro Y (2021). Intake of koji amazake improves defecation frequency in healthy adults. J Fungi.

[94] Li M, Wang B, Zhang M (2008). Symbiotic gut microbes modulate human metabolic phenotypes. Proc Natl Acad Sci.

[95] Wang B, Jiang X, Cao M (2016). Altered fecal microbiota correlates with liver biochemistry in nonobese patients with non-alcoholic fatty liver disease. Sci Rep.

[96] Eun CS, Kwak MJ, Han DS (2016). Does the intestinal microbial community of Korean Crohn's disease patients differ from that of Western patients?. BMC Gastroenterol.

[97] Kasai C, Sugimoto K, Moritani I (2015). Comparison of the gut microbiota composition between obese and non-obese individuals in a Japanese population, as analyzed by terminal restriction fragment length polymorphism and next-generation sequencing. BMC Gastroenterol.

[98] Nam YD, Jung MJ, Roh SW (2011). Comparative analysis of Korean human gut microbiota by barcoded pyrosequencing. PLoS ONE.

[99] Tsai MC, Liu YY, Lin CC (2020). Gut microbiota dysbiosis in patients with biopsy-proven nonalcoholic fatty liver disease: A cross-sectional study in Taiwan. Nutrients.

[100] Chen HM, Yu YN, Wang JL (2013). Decreased dietary fiber intake and structural alteration of gut microbiota in patients with advanced colorectal adenoma. Am J Clin Nutr.

[101] Raethong N, Nakphaichit M, Suratannon N (2021). Analysis of human gut microbiome: Taxonomy and metabolic functions in Thai adults. Genes.

[102] Jain A, Li XH, Chen WN (2018). Similarities and differences in gut microbiome composition correlate with dietary patterns of Indian and Chinese adults. AMB Express.

[103] Ahmad A, Yang W, Chen G (2019). Analysis of gut microbiota of obese individuals with type 2 diabetes and healthy individuals. PLOS ONE.

[104] Shen Y, Xu J, Li Z (2018). Analysis of gut microbiota diversity and auxiliary diagnosis as a biomarker in patients with schizophrenia: A cross-sectional study. Schizophr Res.

[105] Zhuang X, Tian Z, Li L (2018). Fecal microbiota alterations associated with diarrhea-predominant irritable bowel syndrome. Front Microbiol.

[106] Liu F, Fan C, Zhang L (2020). Alterations of gut microbiome in Tibetan patients with coronary heart disease. Front Cell Infect Microbiol.

[107] Oh JH, Lee JH, Cho MS (2021). Characterization of gut microbiome in Korean patients with metabolic associated fatty liver disease. Nutrients.

[108] Zhang YK, Zhang Q, Wang YL (2021). A comparison study of age and colorectal cancer-related gut bacteria. Front Cell Infect Microbiol.

[109] Zhang P, Fang J, Li G (2021). Sex differences in fecal microbiota correlation with physiological and biochemical indices associated with end-stage renal disease caused by immunoglobulin a nephropathy or diabetes. Front Microbiol.

[110] Zhang Z, Zhu L, Ma Y (2022). Study on the characteristics of intestinal flora composition in gastric cancer patients and healthy people in the Qinghai-Tibet plateau. Appl Biochem Biotechnol.

[111] Song ST, Cai LY, Zeng X (2022). Gut microbial profile in asymptomatic gallstones. Front Microbiol.

[112] Ito A, Matsui Y, Takeshita M (2023). Gut microbiota-mediated associations of green tea and catechin intakes with glucose metabolism in individuals without type 2 diabetes mellitus: A four-season observational study with mediation analysis. Arch Microbiol.

[113] Gao M, Wu J, Zhou S (2024). Combining fecal microbiome and metabolomics reveals diagnostic biomarkers for esophageal squamous cell carcinoma. Microbiol Spectr.

[114] Zhu L, Ma Z, Ren M (2020). Distinct features of gut microbiota in high-altitude Tibetan and middle-altitude Han hypertensive patients. Cardiol Res Pract.

[115] Feng Y, Duan Y, Xu Z (2019). An examination of data from the American Gut Project reveals that the dominance of the genus *Bifidobacterium* is associated with the diversity and robustness of the gut microbiota. MicrobiologyOpen.

[116] Blatchford P, Stoklosinski H, Eady S (2017). Consumption of kiwifruit capsules increases *Faecalibacterium prausnitzii* abundance in functionally constipated individuals: A randomised controlled human trial. J Nutr Sci.

[117] McLellan SL, Huse SM, Mueller-Spitz SR (2010). Diversity and population structure of sewage-derived microorganisms in wastewater treatment plant influent. Environ Microbiol.

[118] Shen F, Zheng RD, Sun XQ (2017). Gut microbiota dysbiosis in patients with non-alcoholic fatty liver disease. Hepatobiliary Pancreat Dis Int.

[119] Pinna NK, Anjana RM, Saxena S (2021). Trans-ethnic gut microbial signatures of prediabetic subjects from India and Denmark. Genome Med.

[120] Yeoh YK, Chen Z, Wong MCS (2020). Southern Chinese populations harbour non-nucleatum *Fusobacteria* possessing homologues of the colorectal cancer-associated FadA virulence factor. Gut.

[121] Wang S, Yang L, Hu H (2022). Characteristic gut microbiota and metabolic changes in patients with pulmonary tuberculosis. Microb Biotechnol.

[122] Fontana A, Manchia M, Panebianco C (2020). Exploring the role of gut microbiota in major depressive disorder and in treatment resistance to antidepressants. Biomedicines.

[123] Zheng P, Zeng B, Zhou C (2016). Gut microbiome remodeling induces depressive-like behaviors through a pathway mediated by the host's metabolism. Mol Psychiatry.

[124] Rodríguez-Díaz C, Martín-Reyes F, Taminiau B (2023). The metagenomic composition and effects of fecal-microbe-derived extracellular vesicles on intestinal permeability depend on the patient's disease. Int J Mol Sci.

[125] Ceccarani C, Bassanini G, Montanari C (2020). Proteobacteria overgrowth and butyrate-producing taxa depletion in the gut microbiota of glycogen storage disease type 1 patients. Metabolites.

[126] Yang Y, Du L, Shi D (2021). Dysbiosis of human gut microbiome in young-onset colorectal cancer. Nat Commun.

[127] Granado-Serrano AB, Martín-Garí M, Sánchez V (2019). Faecal bacterial and short-chain fatty acids signature in hypercholesterolemia. Sci Rep.

[128] Glazunova E, Kurnosov A, Zlobovskaya O (2024). Gut dysbiosis and сolorectal cancer: From carcinogenesis hypotheses to non-invasive diagnostics. Bull Russ State Med Univ.

[129] Roesch LFW, Casella G, Simell O (2009). Influence of fecal sample storage on bacterial community diversity. Open Microbiol J.

[130] Gohl DM, Vangay P, Garbe J (2016). Systematic improvement of amplicon marker gene methods for increased accuracy in microbiome studies. Nat Biotechnol.

[131] Tremblay J, Singh K, Fern A (2015). Primer and platform effects on 16S rRNA tag sequencing. Front Microbiol.

[132] Yang B, Wang Y, Qian PY (2016). Sensitivity and correlation of hypervariable regions in 16S rRNA genes in phylogenetic analysis. BMC Bioinformatics.

[133] Zlobovskaya O, Kurnosov A, Sheptulina A (2024). Method for quantitative assesment of gut microbiota: A comparative analysis of 16S NGS and qPCR. Bull Russ State Med Univ.

[134] The Ribosomal RNA Database [Internet].

[135] Jian C, Luukkonen P, Yki-Järvinen H (2020). Quantitative PCR provides a simple and accessible method for quantitative microbiota profiling. PLOS ONE.

