# Supplementary material for: Healthy human gut microbiome: Towards standardized research
Source: AIMS Microbiol. 2025 Nov 4;11(4):786–820. doi: 10.3934/microbiol.2025034 (PMC12783075; doi:10.3934/microbiol.2025034)
Supplement: Supplementary file 1 [file microbiol-11-04-034-s001.pdf]

Microbiology

AIMS Microbiology, 11(4): 786–820.
DOI: 10.3934/microbiol.2025034
Received: 18 July 2025
Revised: 01 October 2025
Accepted: 14 October 2025
Published: 04 November 2025

https://www.aimspress.com/journal/microbiology

Review

Healthy human gut microbiome: Towards standardized research

Evgeniya Glazunova[1], Polina Molodtsova[1], Ilya Grabarnik[1,2], Alexander Kurnosov[1,3], Irina Bikaeva[1], German Shipulin[1] and Olga Zlobovskaya[1,*]

[1] Federal State Budgetary Institution “Centre for Strategic Planning and Management of Biomedical Health Risks” of the Federal Medical and Biological Agency, Russia
[2] Applied Genomics Laboratory, ITMO University, Saint Petersburg, 197101 Russia
[3] N.M. Emmanuel Institute of Biochemical Physics of the Russian Academy of Sciences, Moscow, 119334 Russia

* **Correspondence:** Email: OZlobovskaya@cspfmba.ru; Tel: +7 (495) 5406175 ext. 3035.

Supplementary

Abbreviations: AN: anorexia nervosa; CCS: case-control study; CKD: chronic kidney disease; CRC: colorectal cancer; CS: cohort study; CS0: open-label crossover study; CS1: blind, crossover study; CS2: double-blind, crossover study; DR: diabetic retinopathy; FPG: fasting plasma glucose; FR: fundamental research; HbA1: glycated forms of hemoglobin; IBD: inflammatory bowel disease; IBS: irritable bowel syndrome; NAFLD: non-alcoholic fatty liver disease; NASH: nonalcoholic steatohepatitis; MA: meta-analysis; MMD: myotonic muscular dystrophy; OS: observational study; PBCS: prospective baseline control study; PCS: prospective cohort study; pSS: Sjögren's syndrome, Gougereau's syndrome, dry syndrome, autoimmune exocrinopathy, autoimmune epitheliitis; T1D: type 1 diabetes mellitus; T2D: type 2 diabetes mellitus; UCulcerative colitis

1. Search strategy (keywords)

Our search queries incorporated well-established terms characterizing the microbiome, materials, and research results: Abundance—a quantitative or semi-quantitative measure of the frequency of the sought-after taxon; Relative abundance—an indicator that determines the relative contribution of the desired taxon to the total number of microbiome taxa, most often expressed as a percentage; Beating—mechanical homogenization; Beating beads—grinding particles used for mechanical homogenization; Diversity—the qualitative composition of the taxa included in the ecosystem under consideration.

In accordance with new rules set by the International Committee on Systematics of Prokaryotes, the names of the phyla Firmicutes, Bacteroidetes, Proteobacteria, Actinobacteria, Verrucomicrobia, Fusobacteria, and Tenericutes have been updated to Bacillota, Bacteroidota, Pseudomonadota, Actinomycetota, Verrucomicrobiota, Fusobacteriota, and Mycoplasmatota respectively. Since most studies with key data published to date were expected to follow the previous phyla nomenclature, and given the emergence of newer publications adopting the updated phyla names, we first initially included both the old and new names of the most prevalent phyla term in the search query. Additionally, we ensured that the NCBI Search details incorporated synonyms via MeSH Terms covering both the current phylum nomenclature and its previous indexing heading (e.g., searching for "Firmicutes" as well as its former MeSH heading "Gram-positive Bacteria"). We retain the previous phyla names in this article to connect our results with data from earlier studies and to facilitate communication in research.

The article search strategy included keywords and operators in the following order: (microbiota OR microbiome) and (Firmicutes or Bacteroidetes or Proteobacteria or Actinobacteria OR Verrucomicrobia OR Euryarchaeota OR Tenericutes OR Fusobacteria OR Firmicutes/Bacteroidetes ratio OR Bacillota OR Bacteroidota OR Pseudomonadota OR Actinomycetota OR Verrucomicrobiota OR Fusobacteriota OR Mycoplasmatota OR Firmicutes/Bacteroidetes ratio OR Bacillota/Bacteroidota ratio) AND (human OR homo) AND (healthy OR control) AND DNA extraction AND (colon OR gut OR feces OR fecal OR stool) AND 16S AND (V1 OR V2 OR V3 OR V4 OR V5 OR V6 OR V7 OR V8 OR V9) AND (abundance OR relative abundance OR diversity) NOT animals NOT pregnant NOT (infants OR children).

In addition to the main query, we conducted a specific search using precise keywords and options: When searching for systematic reviews and meta-analyses, we customized the request through the NCBI database dashboard; When searching for family taxa, the query was formulated as follows: (microbiota OR microbiome) AND (Actinomycetaceae OR Akkermansiaceae OR Bacteroidaceae OR Bifidobacteriaceae OR Christensenellaceae OR Clostridiaceae OR Coriobacteriaceae OR Desulfovibrionaceae OR

Enterobacteriaceae OR Enterococcaceae OR Eubacteriaceae OR Fusobacteriaceae OR Lachnospiraceae OR Lactobacillaceae OR Methanobacteriaceae OR Odoribacteraceae OR Oscillospiraceae OR Peptococcaceae OR Peptoniphilaceae OR Peptostreptococcaceae OR Porphyromonadaceae OR Prevotellaceae OR Pseudomonaceae OR Rikenellaceae OR Streptococcaceae OR Tannerellaceae OR Veillonellaceae OR Verrucomicrobiaceae) AND (human OR homo) AND (healthy OR control) AND DNA extraction AND (colon OR gut OR feces OR fecal OR stool) AND 16S AND (V1 OR V2 OR V3 OR V4 OR V5 OR V6 OR V7 OR V8 OR V9) AND (abundance OR relative abundance OR diversity) NOT animals NOT pregnant NOT (infants OR children); When searching for studies without the mechanical homogenization stage, the following keywords were added to the request: NOT (bead OR beating) NOT (PowerSoil OR PowerFecal).

2. Inclusion criteria and exclusion criteria

2.1. Inclusion criteria

1) The publication presents a group of healthy participants aged 18 to 70 years, regardless of gender, race, dietary preferences, or geographic location. The health status of the subjects is defined as the absence of any disease, as determined by medical research or self-completed questionnaires, followed by a medical verdict.
2) Feces were the source for subsequent analysis of microbial DNA.
3) Feces were accepted no later than 2 hours after defecation if stored at room temperature; up to 12 hours at +4 °C; or frozen for long-term storage.
4) A detailed DNA extraction protocol, with or without a mechanical homogenization step, or a link to a well-documented relevant protocol, is provided.
5) 16S rRNA gene sequencing technology was employed for qualitative and quantitative DNA analysis; target regions and the number of reads per sample are indicated.
6) The study conclusion is presented as the relative abundance of taxa, expressed as a percentage, using mean or median values, with or without standard deviation or quartiles.

2.2. Exclusion criteria

1) Recent intake of antibiotics, probiotics, prebiotics, or any other medications that may modulate the qualitative and quantitative composition of the microbiota within at least 1 month prior to the study.
2) For female participants: pregnancy, lactation, or postmenopausal condition.
3) Participants aged less than 18 or over 55 years for women or 70 years for men.
4) Body mass index (BMI)* (sample average) outside the range of 18.5–24.99 kg/m^2. *It is important to note that while overweight or obesity is not classified as a disease, it is considered a risk factor and may correlate with pathological changes in the human intestinal microbiota.
5) The total percentage of identified bacteria and archaea taxa is less than 90%.

3. Ranking is based on the medical evaluation method

1) Medical center examination based on specific criteria, accompanied by clinical laboratory tests.

2) Self-reported examination using a specialized medical questionnaire followed by a medical assessment.
3) Self-reported examination using a specialized medical questionnaire followed by an evaluation by researchers.
4) No data on the examination type.

Among the 86 included studies, 46 articles correspond to rank 1, 13 articles to rank 2, 20 articles to rank 3, and 7 articles to rank 4, which corresponds to 54%, 15%, 23%, and 8%, respectively.

Table S1. Scoring of included studies with homogenisation step.

Western Donors				Eastern Donors			
No	Publication	Rank	Number of participants	No	Publication	Rank	Number of participants
1	Alejandra Chávez-Carbajall, 2019 [1]	1	25	1	Abhishek Jain, 2018 [2]	3	16
2	A B Granado-Serrano, 2019 [3]	1	21	2	Aftab Ahmad, 2019 [4]	4	20
3	Alejandra Chávez-Carbajal, 2020 [5]	1	76	3	Aoi Ito, 2023 [6]	1	81
4	Alexandra Zhernakova, 2016 [7]	2	893	4	Atsushi Kurahashi, 2021 [8]	1	44
5	Aline Zazeri Leite, 2017 [9]	1	22	5	Baohong Wang, 2016 [10]	1	83
6	Andrei Bombin, 2022 [11]	1	76	6	Chang Soo Eun, 2016 [12]	3	15
7	Bruna Stevanato Higuchi, 2018 [13]	2	28	7	Chika Kasai, 2015 [14]	1	23
8	Carlo Bressa, 2017 [15]	2	19/21	8	Feng Shen, 2017 [16]	1	22
9	Christian Benedict, 2016 [17]	2	9	9	Fengyun Liu, 2020 [18]	1	23
10	Cory I Wasser, 2020 [19]	2	36	10	Hui-Min Chen, 2013 [20]	1	47
11	Floris Imhann, 2016 [21]	1	582	11	Joo Hyun Oh, 2021 [22]	1	44
12	Gregory J. Grosicki, 2020 [23]	3	28	12	Junhong Su, 2021 [24]	3	57
13	Ignacio Dei-Cas, 2020 [25]	1	27	13	Lu-lu Zhu, 2020 [26]	1	34
14	Inés Martínez, 2010 [27]	4	10	14	Min Li, 2008 [28]	4	7
15	Julien Tap, 2009 [29]	4	17	15	Ming-Chao Tsai, 2020 [30]	1	25
16	Katja Bezek, 2020 [31]	2	200	16	Mingjun Gao, 2024 [32]	2	20
17	Lauren M. G. Davis, 2011 [33]	4	18	17	Nachon Raethong, 2021 [34]	3	56
18	Manuel Fernandez-Sanjurjo, 2024 [35]	1	15	18	Nishal Kumar Pinna, 2021 [36]	1	137
19	Nadja Larsen, 2010 [37]	3	18	19	P Zheng, 2016 [38]	1	63
20	Natalia González-Zancada, 2020 [39]	2	44/34	20	Peng Zhang, 2021 [40]	1	15
21	Nishal Kumar Pinna, 2021 [36]	1	138	21	Sen-Tao Song, 2022 [41]	1	30
22	Olesya Volokh, 2019 [42]	1	150	22	Xiaojun Zhuang, 2018 [43]	1	13
23	Paul Blatchford, 2017 [44]	2	20	23	Xi-Zhen Sun, 2020 [45]	1	38
24	S.L. McLellan, 2009 [46]	4	25	24	Yang Shen, 2018 [47]	3	53
25	Sergio Ruiz-Saavedra, 2023 [48]	1	20	25	Young-Do Nam, 2011 [49]	3	20
26	Steinar Traae Bjørkhaug, 2019 [50]	1	18	26	Yu-Kun Zhang, 2021 [51]	1	26
27	Tue H Hansen, 2018 [52]	1	29	27	Zilong Zhang, 2021 [53]	1	30
28	Yuqing Feng, 2019 [54]	3	13375				

Table S2. Scoring of included studies without homogenisation step.

Western Donors				Eastern Donors			
No	Publication	Rank	Number of participants	No	Publication	Rank	Number of participants
1	Anastasia Senina, 2024 [55]	1	12	1	Agni Nhirmal Kumar Sugurmar, 2021 [56]	1	12
2	Andrea Fontana, 2020 [57]	1	20	2	Bo Jiao, 2023 [58]	2	27
3	Antonio Cano-Ortiz, 2020 [59]	1	19	3	FengXia Li, 2019 [60]	1	22
4	Camilla Ceccarani, 2020 [61]	1	12	4	Hyuk Yoon, 2023 [62]	3	97
5	Cristina Rodríguez-Díaz, 2023 [63]	4	9	5	Qiangchuan Hou, 2020 [64]	3	106
6	Davide Sisti, 2022 [65]	3	148	6	Sama Kalyana Chakravarthy, 2018 [66]	3	31
7	Elena Barengolts, 2018 [67]	1	24	7	Seok-Won Kim, 2013 [68]	3	18
8	Francesca Borgo, 2017 [69]	1	15	8	Shuting Wang, 2021 [70]	1	52
9	Francesca Gallè, 2020 [71]	3	140	9	Sudhir Kumar, 2024 [72]	2	10
10	Kumar Pallav, 2014 [73]	1	8	10	Sun Min Lee, 2020 [74]	1	12
11	Maria Laura Santoru, 2017 [75]	3	51	11	Taraprasad Das, 2021 [76]	1	30
12	Oliwia Zakerska-Banaszak, 2021 [77]	3	10	12	Vincent Wai-Sun Wong, 2013 [78]	1	22
13	Raylene A Reimer, 2020 [79]	3	13	13	Wanxin Liu, 2020 [80]	3	42
14	Sophie Hiel, 2019 [81]	2	24	14	Xiaofan Yu, 2023 [82]	3	60
				15	Yongzhi Yang, 2019 [83]	1	50
				16	Yongzhi Yang, 2021 [84]	1	100
				17	Yun Kit Yeoh, 2021 [85]	2	78
				18	Zhigang Ren, 2020 [86]	1	210

4. List of included studies

The column “Exclusion Criteria” lists additional exclusion criteria declared by the authors that are not included in the main exclusion list of this meta-analysis (see the section on Screening Stages and Study Quality Assessment).

Table S3. Summary of reviewed studies utilizing preliminary sample homogenization steps involving western donors.

No	Publication	SJR/Quartiles	Study characteristics		Participant characteristics					Extraction		16S rRNA Target Region	Database
			Aim	Study design	Additional exclusion criteria*	Number of participants	Age (mean ± SD, range)	Gender (M/W, n)	Study population and/or cohort location	Feces collection method	Extraction method		
1	Alejandra Chávez-Carbajall, 2019 [1]	1.341/Q1	The study aim was to characterize the faecal microbiota signature and both short chain fatty acids (SCFAs) and bile acids (BA) profile of hypercholesterolemic subjects	CCS	Metabolic pathologies such as T1D or T2D and having taken lipid-lowering medications in last two months, history of high levels of total cholesterol (<200 mg/dL).	21	54.5 ± 7.5	21/0	Caucasian from Lleida, Catalonia, Spain	Collected by participants	Column-based purification method (QIAamp DNA Stool Mini Kit, Qiagen)	V3–V4	Blast, RDP
2	A B Granado-Serrano, 2019 [3]	0.858/Q2	The study determines the differences in gut microbiota in samples of Mexican subjects with different stages of glucose intolerance and healthy control group	CCS	HbA1 ≤ 5.6%, FPG level < 100 mg/dL, vegetarians and vegans, allergies, and autoimmune disease	76	48.0 ± 5.4	50/26	Mexicans	Collected by researchers	Column-based purification method (PowerSoil® DNA Isolation Kit, Qiagen)	V3	Greengenes
3	Alejandra Chávez-Carbajal, 2020 [5]	1.317/Q1	The study identifies alterations of the gut microbiota in samples of healthy Mexican women, women with obesity and women with obesity plus metabolic syndrome	CCS	Smoking, allergies, thyroid disease, consumption of any supplement, and atherosclerotic cardiovascular disease, eating disorders	25	23.3 ± 3.1	0/25	Mexicans	Collected by researchers	Column-based purification method (ZR Fecal DNA MiniPrep™, Zymo Research)	V3	Greengenes, Kyoto Encyclopedia of Genes, KEGG
4	Alexandra Zhernakova, 2016 [7]	13.745/Q1	Deep sequencing of gut microbiomes of participants from a Dutch population-based cohort	PCS	Antifungal and antimalarial drugs intake, steroids, immunosuppressants, antihormones, cytostatics, HIV inhibitors, magnesium, Ursohol	893	44.6 ± 12.9	380/513	Dutch	Collected by participants	Column-based purification method (AllPrep DNA/RNA Mini Kit, Qiagen)	V4	MetaPhlan

Continued on next page

No	Publication	SJR/Quartiles	Study characteristics		Participant characteristics					Extraction		16S rRNA Target Region	Database
			Aim	Study design	Additional exclusion criteria*	Number of participants	Age (mean ± SD, range)	Gender (M/W, n)	Study population and/or cohort location	Feces collection method	Extraction method		
5	Aline Zazeri Leite, 2017 [9]	2.803/Q1	The study evaluates the intestinal dysbiosis in Brazilian T2D patients and its correlation with inflammatory cytokines and lipopolysaccharides (LPS) plasma concentrations	CCS	T2D familial history, vaccination, corticosteroids administration in the last 30 days, presence of surgeries, such as appendectomy, cholecystectomy, and bariatric surgery	22	55.7 ± 8.3	12/10	Sao Paulo, Brazil	Collected by researchers	Column-based purification method (PowerSoil® DNA Isolation Kit, Qiagen)	V3–V4	RDP
6	Andrei Bombin, 2022 [11]	0.789/Q2	The study characterizes oral and gut microbial communities, and evaluates host - microbiota interactions in individuals with different obesity status	CCS	Smoking, medication or supplement use (apart from oral contraceptives)	76	29.6	11/65	USA	Collected by researchers	Column-based purification method (PSP® Spin Stool DNA Plus Kit, Qiagen)	V4	SILVA
7	Bruna Stevanato Higuchi, 2018 [13]	2.021/Q1	The study investigates the intestinal dysbiosis in T1D patients and correlation of these results with clinical parameters and cytokines	CCS	Anti-inflammatories intake, vaccination, and corticosteroids in the last 30 days, gastrointestinal surgeries	28	25.2 ± 9.8	10/18	Sao Paulo, Brazil	Collected by researchers	Column-based purification method (PowerSoil® DNA Isolation Kit, Qiagen)	V3–V4	RDP
8	Carlo Bressa, 2017 [15]	1.164/Q1	The study demonstrates interdependence between some bacterial genera and physical activity	CCS	Previous gastrointestinal surgery, smoking, vegetarian or vegan, nutritional or ergogenic complements	19/21	30.7 ± 5.9/ 32.2 ± 8.7	0/40	Caucasian	Collected by researchers	Column-based purification method (PowerSoil® DNA Isolation Kit, Qiagen)	V3–V4	Greengenes
9	Christian Benedict, 2016 [17]	4.026/Q1	The study conducted in which the impact of sleep loss on the human gut microbiota was assessed, in healthy, young normal-weight individuals.	CS0	Vegan	9	23.3 ± 0.6	9/0	Uppsala, Sweden	Collected by participants	Magnetic beads (MoBio PowerMag® Microbiome kit, USA)	V4	

Continued on next page

No	Publication	SJR/Quartiles	Study characteristics		Participant characteristics					Extraction		16S rRNA Target Region	Database
			Aim	Study design	Additional exclusion criteria*	Number of participants	Age (mean ± SD, range)	Gender (M/W, n)	Study population and/or cohort location	Feces collection method	Extraction method		
10	Cory I Wasser, 2020 [19]	NA	The study characterize the gut microbiome in people with Huntington's disease and determine whether the composition of gut microbiota are significantly related to clinical indicators of disease progression	CCS	Psychiatric illness (except for psychiatric symptoms attributable to Huntington's disease), neurological illness (except for Huntington's disease), a history of traumatic brain injury, recent antibiotic or anti-inflammatory medication use (past 2 months) or current recreational substance use.	36	50.55 (13.90)	15/21	Australia	Collected by participants	Column-based purification method<br><br>(DNeasy® PowerSoil® Pro Kit, Qiagen)	V3–V4	SILVA
11	Floris Imhann, 2016 [21]	7.074/Q1	Case-control analysis of gut microbiota, host genome and clinical phenotypes of IBD	CCS	Antifungal medication, anti-malaria medication, steroids, other immunosuppressants, cytostatics, anti-hormones, opiates, Ursochol, HIV inhibitors, magnesium	582	45.9 ±13.7		Dutch from Northern provinces of the Netherlands	Collected by participants	Column-based purification method<br><br>(AllPrep DNA/RNA Mini Kit, Qiagen)	V4	Greengenes
12	Gregory J. Grosicki, 2020 [23]	1.335/Q1	The study investigates a possible link between self-reported sleep habits and the gut microbiome composition in young healthy individuals	OS	Hypertension (systolic blood pressure ≥ 140 mmHg or diastolic blood pressure ≥ 90 mmHg), sleep medication within past six months	28	29.8 ± 10.4	17/11	Chatham County, Georgia, USA	Collected by participants	Column-based purification method via a liquid-handling robot	V4	
13	Ignacio Dei-Cas, 2020 [25]	1.240/Q1	The study aim was to investigate whether there were differences in the gut microbiota in psoriasis patients vs non-psoriasis controls and between psoriasis severity groups.	CSS	The presence of dermatosis, family history of psoriasis in first degree relatives, immunological disorders, hypertension, fatty liver disease, diabetes mellitus, malignancy, any other serious internal disease, smoking and alcohol abuse.	27	48.7 (18.8)	11/16	Caucasian, Argentine	Collected by participants	Column-based purification method<br><br>(QIAamp® PowerFecal® DNA Kit, Qiagen)	V3–V4	Greengenes

Continued on next page

No	Publication	SJR/Quartiles	Study characteristics		Participant characteristics					Extraction		16S rRNA Target Region	Database
			Aim	Study design	Additional exclusion criteria*	Number of participants	Age (mean ± SD, range)	Gender (M/W, n)	Study population and/or cohort location	Feces collection method	Extraction method		
14	Inés Martínez, 2010 [27]	2.705/Q1	The study investigates an effect of resistant starch types 2 and 4 on the fecal microbial community in human individuals	CS2	Vegetarian diet within three months prior to the start of the study or throughout its duration	10	30.5 ± 7.5	5/5	USA	Collected by researchers	Column-based purification method<br><br>(QIAamp DNA Stool Mini Kit, Qiagen)	V1–V3	RDP
15	Julien Tap, 2009 [29]	2.502/Q1	The study investigates the presence of a set of bacterial species that would be altogether dominant and prevalent within the fecal microbiota of healthy humans	CS		17	41.5 ± 12.5	8/9	France, Netherlands		Precipitation	V3–V8	RDP
16	Katja Bezek, 2020 [31]	1.418/Q1	The study determines a set of parameters that could have a major impact on classifying subjects into a single cluster regarding gut bacteria composition	CS	Endocrine disease, anti-inflammatory drugs or antibiotics 90 days prior to the study	200	35.4 ± 7.0	83/117	Caucasian, Slovenia	Collected by participants	Column-based purification method<br><br>(QIAamp DNA Stool Mini Kit, Qiagen)	V3	RDP, Greengenes
17	Lauren M. G. Davis, 2011 [33]	2.425/Q1	The study gains a community wide perspective of the impact of prebiotic galactooligosaccharide (GOS) on the fecal microbiota of healthy human subjects	CS1	Vegetarian diet within three months prior to the start of the study or throughout its duration	18	19–50	13/5	USA	Collected by researchers	Column-based purification method<br><br>(QIAamp DNA Stool Mini Kit, Qiagen)	V1–V3	RDP
18	Manuel Fernandez-Sanjurjo, 2024 [35]	1.473/Q1	The study investigates the associations between the faecal microbiota composition and SCFA content, performance indicators, dietary intake, and supplement use of professional cyclists	OS		15	30.2 ± 3.4	15/0	predominantly Western Europe	Collected by participants	Column-based purification method<br><br>(E.Z.N.A.® DNA extraction kit, Omega Bio-Tek Ref. D4015-02, Norcross, GA, USA)	V2–4–8/V3–6,7–9	Curated MicroSEQTM 16S Reference Library and Greengenes

Continued on next page

No	Publication	SJR/Quartiles	Study characteristics		Participant characteristics					Extraction		16S rRNA Target Region	Database
			Aim	Study design	Additional exclusion criteria*	Number of participants	Age (mean ± SD, range)	Gender (M/W, n)	Study population and/or cohort location	Feces collection method	Extraction method		
19	Nadja Larsen, 2010 [37]	2.705/Q1	The study assesses differences between the composition of the intestinal microbiota in humans with T2D and non-diabetic persons as control	CCS		18	59 ± 11		Denmark	Collected by participants	Column-based purification method<br><br>(QIAamp DNA Stool Mini Kit, Qiagen)	V4–V6	RDP
20	Natalia González-Zancada, 2020 [39]	0.782/Q2	The study explores the associations between moderate consumption of beer, microbiota composition and short chain fatty acid (SCFA) profile	OS	Chronic liver or lung disease, brain disorders, autoimmune diseases (including thyroid disease), human immunodeficiency virus (HIV), Cushing's syndrome, prescription of chronic medication, any type of special diet	44/34	37.23 ± 5.99/34.05 ± 6.39		Caucasian, Spain	Collected by participants	Column-based purification method<br><br>(QIAamp® DNA Stool, Qiagen)	V3–V4	RDP, Greengenes
21	Nishal Kumar Pinna, 2021 [36]	5.067/Q1	The study investigates a probable association of Megasphaera with impaired glucose tolerance in Indian subjects	CCS		138	51 ± 12	48/90	Danish	Collected by participants	Precipitation	V1–V5	SILVA
22	Olesya Volokh, 2019 [42]	1.329/Q1	The study characterizes changes in the gut microbiota composition after 30 days of oral intake of a yoghurt fortified with *Bifidobacterium animalis subsp. lactis* BB-12	PBCS	Chronic kidney and blood disease, surgical interventions on digestive tract (except appendectomy), allergic diseases within less than four weeks prior to the study initiation, hypertension, more than 10 U of alcohol per week, smoking, specialized nutrition product earlier than three months before the study initiation	150	18–40		Caucasian, Russia	Collected by researchers	Precipitation	V4	Greengenes

Continued on next page

No	Publication	SJR/Quartiles	Study characteristics		Participant characteristics					Extraction		16S rRNA Target Region	Database
			Aim	Study design	Additional exclusion criteria*	Number of participants	Age (mean ± SD, range)	Gender (M/W, n)	Study population and/or cohort location	Feces collection method	Extraction method		
23	Paul Blatchford, 2017 [44]	0.984/Q1	The study investigates the impact of ACTAZIN™ green (2400 and 600 mg) and Livaux™ (2400 mg) gold kiwifruit supplements on fecal microbial composition and metabolites in healthy and functionally constipated (FC) participants	CS2	Vegan, raw food or very high-fiber diet (recommended fiber intake of 25–28 g/day for women and 30–38 g per day for men), renal, pulmonary or neurological disorders, gastroparesis, allergy or sensitivity to kiwifruit, participants who had not met the Rome III criteria	19	38 (from 23 to 56)	2/17	Christchurch, New Zealand	Collected by researchers	Column-based purification method<br><br>(PowerSoil® DNA Isolation Kit, Qiagen)	V3–V4	RDP
24	S.L. McLellan, 2009 [46]	2.502/Q1	The study aim is to profile microbial communities from untreated sewage influent samples	FR		25	28.5 ± 3.5		Mostly Caucasian	Collected by researchers	Column-based purification method<br><br>(QIAamp DNA Stool Mini Kit, Qiagen)	V2, V3, V6	SILVA
25	Sergio Ruiz-Saavedra, 2023 [48]	1.391/Q1	This study explores the changes of the fecal microbiota associated with early intestinal mucosal lesions by considering the morphological alterations of intestinal polyps	CCS	Normal colonoscopy results and confirmation of the absence of mucosal lesions according to histopathological analysis	20	60 ± 9	6/14	Spain (North/North-West)	Collected by participants	Column-based purification method<br><br>(QIAamp® Fast DNA Stool Mini, Qiagen)	V3–V4	SILVA
26	Steinar Traae Bjørkhaug, 2019 [50]	3.245/ Q1	The study examines the gut microbiota composition and its functions in patients with alcohol overconsumption	CCS	Abdominal surgery (except appendectomy), IBD, gastrointestinal cancer, infectious gastroenteritis (last four weeks), cognitive impairment, history of alcohol overconsumption	18	58.2 (34–78)	14/4	Norway	Collected by participants	Precipitation<br><br>(CTAB/SDS method by Novogene)	V3–V4	RDP, GreenGenes

Continued on next page

No	Publication	SJR/Quartiles	Study characteristics		Participant characteristics					Extraction		16S rRNA Target Region	Database
			Aim	Study design	Additional exclusion criteria*	Number of participants	Age (mean ± SD, range)	Gender (M/W, n)	Study population and/or cohort location	Feces collection method	Extraction method		
27	Tue H Hansen, 2018 [52]	1.414/Q1	The study explores a potential impact of electrochemically reduced alkaline drinking water on the human intestinal microbiota and host glucose metabolism	CS0	Previous abdominal surgery, weight stabile for at least two months, habitual consumption of caloric or artificially sweetened beverages in excess of 1 L/week, an average intake of alcohol in excess of 7 units/week, current smokers	29	23.0 (21.0–26.0)	29/0	Denmark	Collected by participants	Column-based purification method<br><br>(NucleoSpin™Soil kit, Macherey-Nagel™ 1 GmbH & Co. KG, Germany)	V4	Greengenes
28	Yuqing Feng, 2019 [54]	1.097/Q2	The study use two large datasets published by the American Gut Project and gut metagenomic dataset to analyze the relationship between *Bifidobacterium* and *Lactobacillus* genera and the community structure of the gut microbiota	MA	Lung and autoimmune diseases	13375	45.18 ± 17.66		USA, United Kingdom	Collected by participants	Column-based purification method<br><br>(PowerSoil® DNA Isolation Kit, Qiagen)	V4	SILVA

Table S4. Summary of reviewed studies utilizing preliminary sample homogenization steps involving eastern donors.

No	Publication	SJR/ Quartiles	Study characteristics		Participant characteristics					Extraction		16S rRNA Target Region	Database
			Aim	Study design	Additional exclusion criteria*	Number of participants	Age (mean ± SD, range)	Gender (M/W, n)	Study population and/or cohort location	Feces collection method	Extraction method		
1	Abhishek Jain, 2018 [2]	0.658/ Q2	The study compares fecal bacterial diversity and composition of healthy Indian and Chinese adults	CS	Smoking, eating, drinking for at least 1–2 h prior to samples collection	5/11	25.8 ± 3.7/27.5 ± 2.4	2/3 7/4	Indian and Chinese, Singapore	Collected by participants	Column-based purification method<br><br>(PowerSoil® DNA Isolation Kit, Qiagen)	V4	RDP, Greengenes
2	Aftab Ahmad, 2019 [4]	1.023/ Q1	The study carries out to understand the gut microbiome and its correlation with individual life style of obese T2D patients in the Pakistani population	CCS		20	37.7 ± 12.1		Punjab Province, Pakistan	Collected by participants	Column-based purification method<br><br>(TIAGEN® DNA Stool kit, Tiangen)	V3–V4	SILVA, Greengenes
3	Aoi Ito, 2023 [6]	0.589/ Q2	This study aims to examine the mediating role of the gut microbiota in the associations between green tea and catechin intakes and glucose metabolism in individuals without T2D	OS		81	43.4	55/26	Japan	Collected by participants	Automatic nucleic acid extractor/Magnetic beads<br><br>(Magtration System 12GC, Precision System Science, Chiba, Japan/FastPrep-24 Instrument, MP Biomedicals, Santa Ana, CA, USA)	V3–V4	TechnoSuruga Lab Microbial Identification database
4	Atsushi Kurahashi, 2021 [8]	0.980/ Q1	The study investigates the impact of koji amazake, a beverage made from steamed rice fermented by *Aspergillus oryzae*, on defecation frequency	CS2	Medical treatment, kidney and blood diseases, alcohol, over-exercising, overeating, and sleeping insufficiently on the day before testing	44	39.7 ± 14.9	18/26	Japan	Collected by participants	Automatic nucleic acid extractor<br><br>(GENE PREP STAR PI-480, Kurabo)	V3–V4	TechnoSuruga Lab Microbial Identification

Continued on next page

No	Publication	SJR/ Quartiles	Study characteristics		Participant characteristics					Extraction		16S rRNA Target Region	Database
			Aim	Study design	Additional exclusion criteria*	Number of participants	Age (mean ± SD, range)	Gender (M/W, n)	Study population and/or cohort location	Feces collection method	Extraction method		
5	Baohong Wang, 2016 [10]	1.692/ Q1	The study investigates the impact of intestinal dysbiosis on non-obese NAFLD and risk of NAFLD progression	CCS	Alcohol consumption (>20 g per day for men and >10 g per day for women), a history of cancer, genetic hemochromatosis respiratory problems, renal diseases, dietary restrictions or lifestyle modifications through diet and exercise at enrollment, a positive smoking history, physical exercise and high-energy food with the 24 hours preceding the examination and sampling	83	42.5 ± 9.5	70/13	Chinese	Collected by participants	Column-based purification method<br><br>(QIAamp DNA Stool Mini Kit, Qiagen)	V3	
6	Chang Soo Eun, 2016 [12]	1.123/ Q1	The study analyzes alteration of the intestinal microbial community structure in Korean Crohn's disease (CD) patients	CCS	History of colon cancer, renal failure	15	35	14/1	Koreans	Collected by participants	Precipitation	V1–V3	RDP
7	Chika Kasai, 2015 [14]	1.043/ Q1	The study investigates relationship between the gut microbiota and obesity	CCS	History of chemotherapy or radiation therapy, and regular use of immunosuppressants (steroids, interferon, etc.)	23	45.6 ± 9.6	11/12	Japan	Collected by researchers	Automatic nucleic acid extractor<br><br>(Precision System Science Co., Ltd.)	V3–V4	TechnoSuruga Lab Microbial Identification
8	Feng Shen, 2017 [16]	0.711/ Q2	The study assesses the contribution of gut microbiota dysbiosis to the pathogenesis of NAFLD	CCS	Excessive alcohol consumption (140 g per week for men, 70 g for women) within the past 12 months, "abnormal" dietary habits (e.g., vegetarian diet)	22	50.5 ± 9.5	17/5	Shanghai, China	Collected by participants	Precipitation	V3–V5	RDP

Continued on next page

No	Publication	SJR/ Quartiles	Study characteristics		Participant characteristics					Extraction		16S rRNA Target Region	Database
			Aim	Study design	Additional exclusion criteria*	Number of participants	Age (mean ± SD, range)	Gender (M/W, n)	Study population and/or cohort location	Feces collection method	Extraction method		
9	Fengyun Liu, 2020 [18]	1.812/ Q1	The study analyzes the alterations of the gut microbiome in CHD patients.	CCS		23	41.5 ± 9.6	10/13	Tibetan native residents, Qinghai Province, China	Collected by researchers	Column-based purification method<br><br>(QIAamp DNA Stool Mini Kit, Qiagen)	V3–V4	Greengenes
10	Hui-Min Chen, 2013 [20]	4.055/ Q1	The study investigates the possible effects of dietary fiber on the fecal microbiota of patients with A-CRA	CCS	Familial adenomatous polyposis, hereditary nonpolyposis colorectal cancer, ulcerative colitis, or Crohn disease, renal, liver or rheumatologic disorders, oral topical corticosteroids	47	57 ± 11	24/23	Shanghai, China	Collected by participants	Precipitation	V1–V3	SILVA
11	Joo Hyun Oh, 2021 [22]	1.287/ Q1	This study aims to identify differences in the gut microbiome between patients with MAFLD and healthy controls in Korean population	CCS	Hypertension or use of antihypertensive medication, noninvasive fibrosis test, low Fibrosis-4 index score	44	51.0 (47.0–55.0)	12/32	Korea	Collected by participants	Column-based purification method<br><br>(UltraClean microbial DNA isolation kit, Mo Bio Laboratories, USA)	V3–V4	EZBioCloud
12	Junhong Su, 2021 [24]	1.833/ Q1	The study establishes the effects of 1 month of intermittent fasting on gut microbiome	PBCS	Hypertension, arthritis, chronic respiratory disorders	30/ 27	18.63 ± 1.75/42.6 ± 7.9	30/0 10/17	Xining city, Qinghai Province, China	Collected by participants	Column-based purification method<br><br>(QIAamp DNA Stool Mini Kit, Qiagen)	V3–V4	Kyoto Encyclopedia of Genes, KEGG

Continued on next page

No	Publication	SJR/ Quartiles	Study characteristics		Participant characteristics					Extraction		16S rRNA Target Region	Database
			Aim	Study design	Additional exclusion criteria*	Number of participants	Age (mean ± SD, range)	Gender (M/W, n)	Study population and/or cohort location	Feces collection method	Extraction method		
13	Lu-lu Zhu, 2020 [26]	0.437/ Q1	The purpose of this study is to define the distinct features of the gut microbiome and their correlation with the pathology of hypertension in mountain areas	OS	Renal failure, stroke, peripheral artery diseases, or any other acute or chronic inflammatory diseases; no use of any drugs, including aspirin, statins, metformin, insulin, or traditional Chinese medicines; abnormal defecation habits, salt intake of all participants < 5 g/day	34	53.2 ± 9.3	18/16	Qinghai-Tibet Plateau, China	Collected by participants	Column-based purification method<br><br>(E.Z.N.A.® DNA extraction kit, Omega Bio-Tek Ref. Norcross, GA, USA)	V3–V4	SILVA
14	Min Li, 2008 [28]	7.034/ Q1	The study investigates four-generation Chinese family to analyze and model the microbial-host metabolic connectivities	CS	Special diets, herbal supplements	7	36.5 ± 18.5	3/4	East China	Collected by participants	Precipitation	full-length 16S	RDP
15	Ming-Chao Tsai, 2020 [30]	1.418/ Q1	The study examines Asian patients with biopsy-proven NAFLD and NASH in terms of dysbiosis of the gut microbiota in NAFLD patients	CCS	Excessive alcohol consumption (>140 g/week in men or >70 g/week in women), history of bariatric surgery, proton pump inhibitors within three months before inclusion, hemochromatosis, Wilson's disease	25	36.7 ± 15.0	12/13	Taiwan	Collected by participants	Column-based purification method<br><br>(QIAamp® PowerFecal® DNA Kit, Qiagen)	V3–V4	KEGG

Continued on next page

No	Publication	SJR/ Quartiles	Study characteristics		Participant characteristics					Extraction		16S rRNA Target Region	Database
			Aim	Study design	Additional exclusion criteria*	Number of participants	Age (mean ± SD, range)	Gender (M/W, n)	Study population and/or cohort location	Feces collection method	Extraction method		
16	Mingjun Gao, 2024 [32]	1.149/ Q1	The study investigates the role of the gut microbiome and its metabolites in the pathogenesis of esophageal squamous cell carcinoma to identify potential biomarkers of early esophageal cancer	CCS	Taking hormones, non-steroidal anti-inflammatory, immunosuppressive drugs, and other drugs that may affect the experimental error within the last 8 weeks, lack of basic clinical information	20	62.5 ± 4.36	15/5	Yangzhou, China	Collected by researchers	Precipitation<br><br>(CTAB method)	V4	SILVA
17	Nachon Raethong, 2021 [34]	1.032/ Q2	The study presents initial research of shotgun metagenomics in a Thai population-based cohort from the middle region of Thailand	CS	Family history of colorectal cancer, allergy to coconut	56	30.0 ± 5.2	43/13	Thai, Thailand	Collected by participants	Column-based purification method<br><br>(QIAamp DNA Stool Mini Kit, Qiagen)	V3–V4	Greengenes
18	Nishal Kumar Pinna, 2021 [36]	5.067/ Q1	The study reports a probable association of Megasphaera with impaired glucose tolerance, which is significantly pronounced in Indian subjects	CCS	Use of medications such as dipeptidyl peptidase-4 inhibitors, acarbose, glucagon-like peptide-1 receptor agonists, and orlistat	137	48 ± 13	63/74	India	Collected by participants	Precipitation	V1–V5	SILVA
19	P Zheng, 2016 [38]	7.176/ Q1	This study demonstrates that dysbiosis of the gut microbiome may have a causal role in the development of depressive-like behaviors	CCS		63	41.8 ± 12.3	23/40	China	Collected by researchers	Column-based purification method<br><br>(PowerSoil® DNA Isolation Kit, Qiagen)	V4–V5	RDP

Continued on next page

No	Publication	SJR/ Quartiles	Study characteristics		Participant characteristics					Extraction		16S rRNA Target Region	Database
			Aim	Study design	Additional exclusion criteria*	Number of participants	Age (mean ± SD, range)	Gender (M/W, n)	Study population and/or cohort location	Feces collection method	Extraction method		
20	Peng Zhang, 2021 [40]	1.314/ Q1	The study compares the differences in the gut microbiota between healthy male and female and patients with end-stage renal disease caused by immunoglobulin	OS		15	48.63 ± 3.52 39.86 ± 2.87	7/8	China	Collected by researchers	Column-based purification method (PowerSoil® DNA Isolation Kit, Qiagen)	V4–V5	RDP
21	Sen-Tao Song, 2022 [41]	1.190/ Q1	The study aims to detect and compare the structural differences of the intestinal microbiota between individuals with and without pathogenesis of asymptomatic gallstones	CCS	No history of traditional Chinese medicines for at least 3 months before sample collection	30	52.58 (7.94)	12/18	Shanghai, China	Collected by participants	Column-based purification method (E.Z.N.A.® DNA extraction kit, Omega Bio-Tek Ref. Norcross, GA, USA)	V3–V4	
22	Xiaojun Zhuang, 2018 [43]	1.633/ Q1	The study characterizes the fecal microbiota alterations in Chinese IBS-D patients and to explore the effect of rifaximin on gut microbiota	CS0	lactose intolerance, renal disease, negative stool culture for pathogenic bacteria, yeast, parasites, and viruses	13	30.54 ± 6.75	8/5	China	Collected by researchers	Column-based purification method (QIAamp® PowerFecal® DNA Kit, Qiagen)	V3–V4	RDP
23	Xi-Zhen Sun, 2020 [45]	1.427/ Q1	The study investigates features of tryptophan metabolism in CRC and correlation between fecal tryptophan metabolites and gut microbiota	CCS	History of psychiatric disorders, nonsteroidal anti-inflammatory drugs, immune modulators, corticosteroids, prokinetics, or antispasmodics within 4 week	38	56.85 ± 10.99	24/14	China	Collected by researchers	Column-based purification method (QIAamp® Fast DNA Stool Mini, Qiagen)	V3–V4	

Continued on next page

No	Publication	SJR/ Quartiles	Study characteristics		Participant characteristics					Extraction		16S rRNA Target Region	Database
			Aim	Study design	Additional exclusion criteria*	Number of participants	Age (mean ± SD, range)	Gender (M/W, n)	Study population and/or cohort location	Feces collection method	Extraction method		
24	Yang Shen, 2018 [47]	2.168/ Q1	The study compares the difference in gut microbiota between schizophrenia patients and healthy controls	CCS	Any specific drug intake for the latest 6 months, including glucocorticoids, cytokines, presence of any major surgery with gastrointestinal tract within 5 years, psychiatric symptoms, significant changing in dietary habits and middle or high doses of alcohol abuse or dependence, absence of gastroscopy, colonoscopy, hypertension, immunodeficiency, autoimmune diseases	53	39 ± 14	35/18	Han from Huludao, Northeast China	Collected by researchers	Column-based purification method<br><br>(PowerSoil® DNA Isolation Kit, Qiagen)	V3–V4	Greengenes
25	Young-Do Nam, 2011 [49]	2.425/ Q1	The study investigates the overall composition of gut microbiota and its host specificity and temporal stability in Koreans	CS		20	42 ± 20.3	12/8	Koreans, Republic of Korea	Collected by participants	Precipitation	V1–V3	Greengenes
26	Yu-Kun Zhang, 2021 [51]	1.389/ Q1	This study profiles the intestinal microbiota of young and older volunteers to evaluate how age-related differences should be considered when using gut bacteria as biomarkers for CRC	CCS	Individuals with a special diet; individuals who received fecal microbiota transplantation therapy	26	21–26	19/7	Harbin, China	Collected by researchers	Column-based purification method<br><br>E.Z.N.A.® DNA extraction kit (Omega Bio-Tek Ref. D4015-02, Norcross, GA, USA)	V3–V4	SILVA

Continued on next page

No	Publication	SJR/ Quartiles	Study characteristics		Participant characteristics					Extraction		16S rRNA Target Region	Database
			Aim	Study design	Additional exclusion criteria*	Number of participants	Age (mean ± SD, range)	Gender (M/ W, n)	Study population and/or cohort location	Feces collection method	Extraction method		
27	Zilong Zhang, 2021 [53]	0.475/ Q2	The study compares and analyzes the structure and diversity of the intestinal flora between gastric cancer patients and healthy people in the Qinghai-Tibet Plateau	CCS	Radiotherapy, and chemotherapy in the past	30	58.87 ± 15.520	15/15	Qinghai Province, China	Collected by participants	Magnetic beads<br><br>(MagPure Soil DNA Kit Magen Biotechnology Co., Ltd,China)	V3–V4	GreenGenes, SILVA, RDP

Table S5. Summary of reviewed studies without preliminary sample homogenization steps involving western donors.

No	Publication	SJR/Quartiles	Study characteristics		Participant characteristics					Extraction		16S rRNA Target Region	Database
			Aim	Study design	Additional exclusion criteria*	Number of participants	Age (mean ± SD, range)	Gender (M/ W, n)	Study population and/or cohort location	Study population and/or cohort location	Feces collection method		
1	Anastasia Senina, 2024 [55]	1.009/ Q2	This study investigates long-term changes in the gut microbiota composition and SCFA production in healthy volunteers in the Volga region of Russia, including assessment of the seasonal effects	OS		12	26–52	13/7	Russia	Collected by participants	Column-based purification method<br><br>(FastDNA ™ SPIN Kit for Feces, Biomedicals, CA, USA)	V3–V4	Greengenes

Continued on next page

No	Publication	SJR/Quartiles	Study characteristics		Participant characteristics					Extraction		16S rRNA Target Region	Database
			Aim	Study design	Additional exclusion criteria*	Number of participants	Age (mean ± SD, range)	Gender (M/W, n)	Study population and/or cohort location	Study population and/or cohort location	Feces collection method		
2	Andrea Fontana, 2020 [57]	1.511/ Q1	The study aims to explore potential differences in the composition of the gut microbiota between patients with MDD and healthy controls to identify possible signatures of treatment response	CSS	History of mental disorders, the willingness to participate in the study, absence of acute infections; absence of chronic autoimmune inflammatory conditions (eg, rheumatoid arthritis and thyroiditis); absence of past traumatic brain injury;	20	37.7 [30.6–58.0]	13/7	Italy	Collected by participants	Column-based purification method (QIAamp DNA Stool Mini Kit, Qiagen)	V3–V4	Custom databases (based on NCBI)
3	Antonio Cano-Ortiz, 2020 [59]	1.455/ Q1	The study explores intestinal microbial composition and functionality in primary Sjögren's syndrome (pSS) and relates these findings to inflammation, permeability and transcription factor Forkhead box protein P3 (FOXP3) gene expression in peripheral blood	CCS		19	54.70 ± 8.42	0/19	Spain		Column-based purification method (QIAamp DNA Stool Mini Kit, Qiagen)	V2–V9	Greengenes
4	Camilla Ceccarani, 2020 [61]	1.109/ Q2	The study assesses the impact of the dietary treatment by comparing the gut microbiota of GSD-I patients and healthy controls	CCS	Gstational age 37–42 week inclusive, birth weight ≥ 2500 g and single birth;	12	24.7 ± 7.9	9/13	Italy	Collected by researchers	Spin stool DNA kit (Stratec Molecular, Berlin, Germany)	V3–V4	Greengenes

Continued on next page

No	Publication	SJR/Quartiles	Study characteristics		Participant characteristics					Extraction		16S rRNA Target Region	Database
			Aim	Study design	Additional exclusion criteria*	Number of participants	Age (mean ± SD, range)	Gender (M/W, n)	Study population and/or cohort location	Study population and/or cohort location	Feces collection method		
5	Cristina Rodríguez-Díaz, 2023 [63]	1.179/ Q1	The study explores metagenomic profiling of feces and fecal-microbe-derived extracellular vesicles from healthy subjects and patients with different gut-associated disorders, as well as their effect on Caco-2 cell.	OS		9			Caucasian, Spain	Collected by researchers	Column-based purification method (DNeasy blood and Tissue Kits, Qiagen)	V1–V3	SILVA
6	Davide Sisti, 2022 [65]	0.973/ Q1	The study identifies the existence of a primary healthy microbiological framework that is only marginally affected by variations in healthy Italian cohort	CS	Autoimmune or psychiatric disorders	148	18–65	69/79	Caucasian, Italy	Collected by participants	Column-based purification method (QIAamp DNA Stool Mini Kit, Qiagen)	V3–V4	SILVA
7	Elena Barengolts, 2018 [67]	1.100/ Q1	The study tests the hypothesis that variability in gut microbiota in T2D is associated with psycho-metabolic health	CCS	Chronic kidney disease (stages 3b, 4, and 5), chronic glucocorticoid intake (3 months or longer), non-metformin antihyperglycemics intake, and presence or history of significant health conditions requiring recent (within 6 months) hospitalization	24	54.6 ± 7.3	24/0	African Americans	Collected by researchers	Automatic nucleic acid extractor (Maxwell® 16 Tissue DNA Purification Kit, Promega)	V3–V4	Greengenes

Continued on next page

No	Publication	SJR/Quartiles	Study characteristics		Participant characteristics					Extraction		16S rRNA Target Region	Database
			Aim	Study design	Additional exclusion criteria*	Number of participants	Age (mean ± SD, range)	Gender (M/W, n)	Study population and/or cohort location	Study population and/or cohort location	Feces collection method		
8	Francesca Borgo, 2017 [69]	1.164/ Q1	The study performs a comprehensive analysis integrating data on gut microbiota with clinical, anthropometric and psychological traits to gain new insight in the pathophysiology of AN	CCS	Celiac disease, history of colorectal cancer, binge eating or purging behavior, recent enteral/parenteral nutrition	15	24.4 ± 3.9	0/15	Italy	Collected by researchers	Column-based purification method<br><br>(PSP ® Spin Stool DNA Kit, lnvitek Molecular)	V3, V4	
9	Francesca Gallè, 2020 [71]	1.418/ Q1	The study explores the microbial composition of the gut and its possible association with the Mediterranean diet	CCS	Food intolerances	140	22.5 ± 2.9	68/72	Italy	Collected by participants	Column-based purification method<br><br>(QIAamp DNA Stool Mini Kit, Qiagen)	V1–V2	RDP, Greengenes
10	Kumar Pallav, 2014 [73]	1.705/ Q1	The study compares the effects of the prebiotic, polysaccharopeptide from *Trametes versicolor*, to those of the antibiotic, amoxicillin, on the human gut microbiome	CS0	Autoimmune disease, history of organ transplantation, prior gastrointestinal surgery (apart from appendectomy or cholecystectomy), history of allergy or hypersensitivity to mushroom or mushroom extract, brewer's or baker's yeast or to penicillins or cephalosporins, medications during the 4 weeks prior to study enrollment	8	29.9 ± 10.3	3/5	USA	Collected by participants	Column-based purification method<br><br>(QIAamp DNA Stool Mini Kit, Qiagen)	V1–V3	RDP

Continued on next page

No	Publication	SJR/Quartiles	Study characteristics		Participant characteristics					Extraction		16S rRNA Target Region	Database
			Aim	Study design	Additional exclusion criteria*	Number of participants	Age (mean ± SD, range)	Gender (M/W, n)	Study population and/or cohort location	Study population and/or cohort location	Feces collection method		
11	Maria Laura Santoru, 2017 [75]	1.533/ Q1	The study underlines the potential role of an inter-omics approach in understanding the metabolic pathways involved in IBD	CCS		51	56.85 ± 10.99	31/20	Italy	Collected by participants	Column-based purification method<br><br>(QIAamp DNA Stool Mini Kit, Qiagen)	V3, V4	Greengenes
12	Oliwia Zakerska-Banaszak, 2021 [77]	1.005/ Q1	The study determines specific changes in the gut microbial profile in Polish UC patients compared to healthy subjects	CCS	Alcohol abuse during the last 6 months	10	45.2 ± 9.3	4/6	Poles	Collected by researchers	Automatic precipitation<br><br>(Genomic Mini AX Bacteria, A&A Biotechnolog)	V3–V4	SILVA
13	Raylene A Reimer, 2020 [79]	2.608/ Q1	The study examines the effect of low and moderate-dose fiber-containing snack bars, comprising mainly chicory root inulin-type fructans, on the gut microbiota.	CS2	Consumption >15 g fiber/d in women and >18 g fiber/d in men, T1D or T2D, cardiovascular abnormalities, liver or pancreas disease, major GI surgeries including bowel resection or gastric bypass, exhibited alcohol or drug dependence; were following a diet, exercise, drug, or supplement regime designed for weight loss	13	30.5 ± 8.6	2/11	Canada	Collected by participants	Column-based purification method<br><br>(FastDNA ™ SPIN Kit for Feces, Biomedicals, CA, USA)	V3–V4	

Continued on next page

No	Publication	SJR/Quartiles	Study characteristics		Participant characteristics					Extraction		16S rRNA Target Region	Database
			Aim	Study design	Additional exclusion criteria*	Number of participants	Age (mean ± SD, range)	Gender (M/W, n)	Study population and/or cohort location	Study population and/or cohort location	Feces collection method		
14	Sophie Hiel, 2019[81]	2.704/ Q1	The study evaluates the impact of consuming ITF-rich vegetables daily on gut microbiota, gastro-intestinal symptoms and food-related behavior in healthy individuals	CS	Level of expired H_2 > 12 ppm, smoking, psychiatric problems, special diet (e.g., vegetarian, high-fiber, or high-protein diets), and excessive alcohol consumption (more than 3 units/day)	24	21.84 ± 0.39	11/13	Caucasian, Belgium	Collected by participants	Column-based purification method<br><br>(PSP ® Spin Stool DNA Kit, lnvitek Molecular)	V5–V6	RDP

Table S6. Summary of reviewed studies without preliminary sample homogenization steps involving eastern donors.

No	Publication	SJR/Quartiles	Study characteristics		Participant characteristics					Extraction		16S rRNA Target Region	Database
			Aim	Study design	Additional exclusion criteria*	Number of participants	Age (mean ± SD, range)	Gender (M/W, n)	Study population and/or cohort location	Study population and/or cohort location	Feces collection method		
1	Agni Nhirmal Kumar Sugurmar, 2021 [56]	0.759/ Q2	The study compares the gut microbial abundance and diversity between healthy volunteers and Malaysian IgAN cohort	CCS	Serum urea more than 20 mmol/L, autoimmune diseases, participants who have travelled abroad in the past 3 months prior to the study	12	46.5 ± 13.5	4/8	Malays, Chinese	Collected by participants	Column-based purification method<br><br>(GeneAll Exgene™ Stool DNA kit, GeneAll)	V3–V4	RDP

Continued on next page

No	Publication	SJR/Quartiles	Study characteristics		Participant characteristics					Extraction		16S rRNA Target Region	Database
			Aim	Study design	Additional exclusion criteria*	Number of participants	Age (mean ± SD, range)	Gender (M/W, n)	Study population and/or cohort location	Study population and/or cohort location	Feces collection method		
2	Bo Jiao, 2023 [58]	0.726/ Q2	The study compares levels of bacterial richness and diversity in the gut microbiota of Postherpetic Neuralgia patients and healthy controls	CSS	An autoimmune component (such as rheumatoid arthritis), intestinal dysfunction, abnormal liver and kidney function, pain medications, anti-inflammatory drugs, antibiotics, or psychotropic medications in the last 6 months	27	55.70 ± 15.384	15/12	Hubei, China	Collected by participants	Precipitation<br><br>(CTAB method)	V3–V4	SILVA
3	FengXia Li, 2019 [60]	1.626/ Q1	The study conductes 16S rDNA pyrosequencing using the fecal microbiota samples and analyzes the production of serum inflammatory factors in patients with CKD compared to healthy subjects	CCS		22	50.27 ± 7.77	12/10	China	Collected by participants	Column-based purification method<br><br>(QIAamp DNA Stool Mini Kit, Qiagen)	V3–V4	RDP
4	Hyuk Yoon, 2023 [62]	0.944/ Q2	This study aims to evaluate the bacterial and fungal biomarkers to differentiate patients with IBD	CCS		97	36 (15–71)	74/23	Korea	Collected by participants	Automatic nucleic acid extractor<br><br>(Maxwell® RSC PureFood GMO and Authentication Kit Promega, Republic of Korea)	V3–V4	RDP

Continued on next page

No	Publication	SJR/Quartiles	Study characteristics		Participant characteristics					Extraction		16S rRNA Target Region	Database
			Aim	Study design	Additional exclusion criteria*	Number of participants	Age (mean ± SD, range)	Gender (M/W, n)	Study population and/or cohort location	Study population and/or cohort location	Feces collection method		
5	Qiangchuan Hou, 2020 [64]	3.294/ Q1	The study studies the effect of taking of the probiotic *Lactobacillus casei* Zhang (LCZ) by healthy adults from six different Asian regions	PBCS	Antibiotics in the 6 months prior to the experiment	106	22.6 ± 2.7		Mongolia, Inner Mongolia, Xinjiang Gansu, Singapore, Indonesia	Collected by participants	Column-based purification method (QIAamp DNA Stool Mini Kit, Qiagen)	full-length 16 S	RDP, Greengenes, SILVA
6	Sama Kalyana Chakravarthy, 2018 [66]	1.100/ Q1	The study demonstrates dysbiosis in the gut bacterial microbiomes of fungal keratitis patients compared to healthy controls	CCS		31	21–81	15/16	India	Collected by participants	Column-based purification method (QIAamp DNA Stool Mini Kit, Qiagen)	V3–V4	Greengenes
7	Seok-Won Kim, 2013 [68]	1.738/ Q1	The study comprehensively investigates effects of probiotics on human intestinal microbiota	PBCS		18	22 ± 3.16	6/12	Japan	Collected by researchers	Precipitation	V1–V2	DDBJ, GenBank, EMBL
8	Shuting Wang, 2021 [70]	0.569/ Q2	The study describes the imbalance of the gut microbiota and altered faecal metabolomics profiles in patients with Pulmonary Tuberculosis	OS	Negative T-SPOT.TB tests	52	32 (26–37)	21/31	China	Collected by researchers	Column-based purification method (QIAamp DNA Stool Mini Kit, Qiagen)	V3–V4	SILVA
9	Sudhir Kumar, 2024 [72]	1.032/ Q1	This study reports the first data from a non-western cohort of vitiligo patients from India	CCS	Complaints of vitiligo or skin depigmentation	10	36.3 (13.4)	6/4	Madhya Pradesh, India	Collected by participants	Column-based purification method (QIAamp Fast DNA Stool Mini Kit, Qiagen)	V3	Greengenes

Continued on next page

No	Publication	SJR/Quartiles	Study characteristics		Participant characteristics					Extraction		16S rRNA Target Region	Database
			Aim	Study design	Additional exclusion criteria*	Number of participants	Age (mean ± SD, range)	Gender (M/W, n)	Study population and/or cohort location	Study population and/or cohort location	Feces collection method		
10	Sun Min Lee, 2020 [74]	1.331/Q1	This study aims to characterize the changes in the gut microbiota of IBS patients	CCS	History of IBD, abdominal operation, severe systemic disease, and malignancy	12	46.1–52.7	4/8	Korea	Collected by participants	Column-based purification method (QIAamp DNA Stool Mini Kit, Qiagen)	V3–V4	Database from EzBioCloud
11	Tarapras ad Das, 2021 [76]	1.005/Q1	The study explores possible link between gut bacterial microbiome dysbiosis and T2DM and DR	CCS	Steroid intake within 90 days, intraocular surgery or intra-vitreal injections, uncontrolled glaucoma, presence of any form of ocular malignancy, kidney disease	30	59.5	17/13	Telingana State, Andhra Pradesh State, India	Collected by participants	Column-based purification method (QIAamp DNA Stool Mini Kit, Qiagen)	V3–V4	Greengenes
12	Vincent Wai-Sun Wong, 2013 [78]	1.772/Q1	The study characterizes the fecal microbiota in patients with NASH	CS0	Alcohol consumption (over 20 g per day for men or 10 g per day for women), use of systemic steroids or methotrexate	22	44 ± 10	9/13	China	Collected by researchers	Column-based purification method (QIAamp DNA Stool Mini Kit, Qiagen)	V1–V2	RDP
13	Wanxin Liu, 2020 [80]	0.772/Q2	The goal of this study is to determine the role of the gut microbiome in the initiation and development of CRC	CCS	Antibiotics or microecological agents within 2 months, intestinal infection or digestive tract symptoms, hypertension, heart disease, diabetes, history of CRC, IBD, or IBS	42	50–70		China	Collected by participants	Column-based purification method (QIAamp DNA Stool Mini Kit, Qiagen)	V4	RDP, Greengenes
14	Xiaofan Yu, 2023 [82]	1.285/Q1	The study aims to identify the particular bacteria that cause MMD	CCS	Without occlusive cerebrovascular disease by MRI, regular medical examinations Smoking 1	60	38.93 ± 10.33	23/37	Beijing, China	Collected by participants	Precipitation (CTAB method)	V3–V4, V4–V5, V9	SILVA

Continued on next page

No	Publication	SJR/Quartiles	Study characteristics		Participant characteristics					Extraction		16S rRNA Target Region	Database
			Aim	Study design	Additional exclusion criteria*	Number of participants	Age (mean ± SD, range)	Gender (M/W, n)	Study population and/or cohort location	Study population and/or cohort location	Feces collection method		
15	Yongzhi Yang, 2019 [83]	2.348/ Q1	The study explores evidence of a novel interplay between the gut microbiome and metabolome, which is drastically perturbed in CRC.	CCS	Younger than 25 years, history of familial CRC, IBD, or IBS, preoperative neoadjuvant chemoradiotherapy, coexisting malignant tumors, complete intestinal obstruction	50	25–80	17/33	Han from Shanghai, China	Collected by participants	Column-based purification method<br><br>(QIAamp DNA Stool Mini Kit, Qiagen)	V3–V4	RDP
16	Yongzhi Yang, 2021 [84]	4.846/ Q1	This study highlights the potential of the gut microbiota biomarkers as a promising non-invasive tool for the accurate detection and distinction of individuals with young-onset CRC	CS0		100	63.23 ± 8.56 39.76 ± 6.11	48/52	Fudan, Huadong, China	Collected by researchers	Column-based purification method<br><br>(QIAamp DNA Stool Mini Kit, Qiagen)	V3, V4	SILVA
17	Yun Kit Yeoh, 2021 [85]	7.885/ Q1	The study explores how the gut microbiome is linked to disease severity in patients with COVID-19.	CS	Solid organ or stem cell transplantation, HIV-positiveness with detectable viral load, prednisone > 0.5 mg/kg, immunomodulatory treatment or recent chemotherapy	78	45.5 ± 13.3	33/45	Southern Chinese, Hong Kong	Collected by participants	Automatic nucleic acid extractor<br><br>(Maxwell® RSC PureFood GMO and Authentication Kit Promega, Wisconsin, USA)		NCBI, (HR) database, GTDB
18	Zhigang Ren, 2020 [86]	5.388/ Q1	The study explores the gut microbiome in patients with chronic kidney disease	CS1		210	50.02 ± 4.56	105/105	Zhengzhou, China	Collected by researchers	Column-based purification method<br><br>(QIAamp DNA Stool Mini Kit, Qiagen)	V3–V4	16S “golden standard” database provided by Broad Institute

Table S7. Excluded studies: 11 publications were excluded at full-text screening stage based on the data expression.

No	Publication	Brief reasons
1	Gabriella C van Zanten, 2014 [87]	data expressed by the median
2	Gózd-Barszczewska A., 2017 [88]	data expressed by the median
3	Jiachao Zhang, 2014 [89]	data expressed by the median
4	Julio Plaza-Díaz, 2015 [90]	data expressed by the median
5	Lisa Lindheim, 2017 [91]	data expressed by the median
6	Marc Clos-Garcia, 2020 [92]	data expressed by the median
7	Naofumi Yoshida, 2022 [93]	data expressed by the median
8	Orawan La-ongkham, 2020 [94]	data expressed by the median
9	Tingting Wang, 2012 [95]	data expressed by the median
10	Toshitaka Odamaki, 2016 [96]	data expressed by the median
11	Zhang Mingjun, 2022 [97]	authors did not indicate statistical parameter for the published data
12	Barbara Zapała, 2021 [98]	data expressed by the median
13	Ling Zhou, 2019 [99]	data expressed by the median
14	Gyeol Seong, 2020 [100]	data expressed by the median
15	Julia K. Copeland, 2021 [101]	data expressed by the median
16	Telma Caleça, 2023 [102]	data expressed by the median
17	María Esteban-Torres 2018 [103]	no *Bacteroidetes* reads identified
18	Jagadesan Sankarasubramanian, 2020 [104]	inclusion/exclusion criteria for controls not reported

References

1. Chávez-Carbajal A, Nirmalkar K, Pérez-Lizaur A, et al. (2019) Gut microbiota and predicted metabolic pathways in a sample of mexican women affected by obesity and obesity plus metabolic syndrome. *Int J Mol Sci* 20: 438. https://doi.org/10.3390/ijms20020438
2. Jain A, Li XH, Chen WN (2018) Similarities and differences in gut microbiome composition correlate with dietary patterns of Indian and Chinese adults. *AMB Express* 8: 104. https://doi.org/10.1186/s13568-018-0632-1
3. Granado-Serrano AB, Martín-Garí M, Sánchez V, et al. (2019) Faecal bacterial and short-chain fatty acids signature in hypercholesterolemia. *Sci Rep* 9. https://doi.org/10.1038/s41598-019-38874-3
4. Ahmad A, Yang W, Chen G, et al. (2019) Analysis of gut microbiota of obese individuals with type 2 diabetes and healthy individuals. *PLOS ONE* 14: e0226372. https://doi.org/10.1371/journal.pone.0226372
5. Chávez-Carbajal A, Pizano-Zárate ML, Hernández-Quiroz F, et al. (2020) Characterization of the gut microbiota of individuals at different T2D stages reveals a complex relationship with the host. *Microorganisms* 8: 94. https://doi.org/10.3390/microorganisms8010094

6. Ito A, Matsui Y, Takeshita M, et al. (2023) Gut microbiota-mediated associations of green tea and catechin intakes with glucose metabolism in individuals without type 2 diabetes mellitus: A four-season observational study with mediation analysis. *Arch Microbiol* 205: 191. https://doi.org/10.1007/s00203-023-03522-y
7. Zhernakova A, Kurilshikov A, Bonder MJ, et al. (2016) Population-based metagenomics analysis reveals markers for gut microbiome composition and diversity. *Science* 352: 565–569. https://doi.org/10.1126/science.aad3369
8. Kurahashi A, Enomoto T, Oguro Y, et al. (2021) Intake of *Koji Amazake* improves defecation frequency in healthy adults. *J Fungi* 7: 782. https://doi.org/10.3390/jof7090782
9. Leite AZ, Rodrigues N de C, Gonzaga MI, et al. (2017) Detection of increased plasma interleukin-6 levels and prevalence of *Prevotella copri* and *Bacteroides vulgatus* in the feces of type 2 diabetes patients. *Front Immunol* 8. https://doi.org/10.3389/fimmu.2017.01107
10. Wang X, Wang J, Rao B, et al. (2017) Gut flora profiling and fecal metabolite composition of colorectal cancer patients and healthy individuals. *Exp Ther Med* 13: 2848–2854. https://doi.org/10.3892/etm.2017.4367
11. Bombin A, Yan S, Bombin S, et al. (2022) Obesity influences composition of salivary and fecal microbiota and impacts the interactions between bacterial taxa. *Physiol Rep* 10. https://doi.org/10.14814/phy2.15254
12. Eun CS, Kwak M-J, Han DS, et al. (2016) Does the intestinal microbial community of Korean Crohn's disease patients differ from that of western patients? *BMC Gastroenterol* 16. https://doi.org/10.1186/s12876-016-0437-0
13. Higuchi BS, Rodrigues N, Gonzaga MI, et al. (2018) Intestinal dysbiosis in autoimmune diabetes is correlated with poor glycemic control and increased interleukin-6: A pilot study. *Front Immunol* 9. https://doi.org/10.3389/fimmu.2018.01689
14. Kasai C, Sugimoto K, Moritani I, et al. (2015) Comparison of the gut microbiota composition between obese and non-obese individuals in a Japanese population, as analyzed by terminal restriction fragment length polymorphism and next-generation sequencing. *BMC Gastroenterol* 15. https://doi.org/10.1186/s12876-015-0330-2
15. Bressa C, Bailén-Andrino M, Pérez-Santiago J, et al. (2017) Differences in gut microbiota profile between women with active lifestyle and sedentary women. *PLOS ONE* 12: e0171352. https://doi.org/10.1371/journal.pone.0171352
16. Shen F, Zheng RD, Sun XQ, et al. (2017) Gut microbiota dysbiosis in patients with non-alcoholic fatty liver disease. *Hepatobiliary Pancreat Dis Int* 16: 375–381. https://doi.org/10.1016/S1499-3872(17)60019-5
17. Benedict C, Vogel H, Jonas W, et al. (2016) Gut microbiota and glucometabolic alterations in response to recurrent partial sleep deprivation in normal-weight young individuals. *Mol Metab* 5: 1175–1186. https://doi.org/10.1016/j.molmet.2016.10.003
18. Liu X, Cheng Y, Shao L, et al. (2020) Alterations of the predominant fecal microbiota and disruption of the gut mucosal barrier in patients with early-stage colorectal cancer. *BioMed Res Int* 2020. https://doi.org/10.1155/2020/2948282
19. Wasser CI, Mercieca EC, Kong G, et al. (2020) Gut dysbiosis in Huntington's disease: Associations among gut microbiota, cognitive performance and clinical outcomes. *Brain Commun* 2: fcaa110. https://doi.org/10.1093/braincomms/fcaa110

20. Chen HM, Yu YN, Wang JL, et al. (2013) Decreased dietary fiber intake and structural alteration of gut microbiota in patients with advanced colorectal adenoma. *Am J Clin Nutr* 97: 1044–1052. https://doi.org/10.3945/ajcn.112.046607
21. Imhann F, Vich Vila A, Bonder MJ, et al. (2018) Interplay of host genetics and gut microbiota underlying the onset and clinical presentation of inflammatory bowel disease. *Gut* 67: 108–119. https://doi.org/10.1136/gutjnl-2016-312135
22. Oh JH, Lee JH, Cho MS, et al. (2021) Characterization of gut microbiome in Korean patients with metabolic associated fatty liver disease. *Nutrients* 13: 1013. https://doi.org/10.3390/nu13031013
23. Grosicki GJ, Riemann BL, Flatt AA, et al. (2020) Self-reported sleep quality is associated with gut microbiome composition in young, healthy individuals: A pilot study. *Sleep Med* 73: 76–81. https://doi.org/10.1016/j.sleep.2020.04.013
24. Su J, Wang Y, Zhang X, et al. (2021) Remodeling of the gut microbiome during Ramadan-associated intermittent fasting. *Am J Clin Nutr* 113: 1332–1342. https://doi.org/10.1093/ajcn/nqaa388
25. Dei-Cas I, Giliberto F, Luce L, et al. (2020) Metagenomic analysis of gut microbiota in non-treated plaque psoriasis patients stratified by disease severity: development of a new Psoriasis-Microbiome Index. *Sci Rep* 10: 12754. https://doi.org/10.1038/s41598-020-69537-3
26. Zhu L, Ma Z, Ren M, et al. (2020) Distinct features of gut microbiota in high-altitude tibetan and middle-altitude han hypertensive patients. *Cardiol Res Pract* 2020: 1–15. https://doi.org/10.1155/2020/1957843
27. Martínez I, Kim J, Duffy PR, et al. (2010) Resistant starches types 2 and 4 have differential effects on the composition of the fecal microbiota in human subjects. *PLoS ONE* 5: e15046. https://doi.org/10.1371/journal.pone.0015046
28. Li M, Wang B, Zhang M, et al. (2008) Symbiotic gut microbes modulate human metabolic phenotypes. *Proc Natl Acad Sci* 105: 2117–2122. https://doi.org/10.1073/pnas.0712038105
29. Tap J, Mondot S, Levenez F, et al. (2009) Towards the human intestinal microbiota phylogenetic core. *Environ Microbiol* 11: 2574–2584. https://doi.org/10.1111/j.1462-2920.2009.01982.x
30. Tsai MC, Liu YY, Lin CC, et al. (2020) Gut microbiota dysbiosis in patients with biopsy-proven nonalcoholic fatty liver disease: A cross-sectional study in Taiwan. *Nutrients* 12: 820. https://doi.org/10.3390/nu12030820
31. Bezek K, Petelin A, Pražnikar J, et al. (2020) Obesity measures and dietary parameters as predictors of gut microbiota phyla in healthy individuals. *Nutrients* 12: 2695. https://doi.org/10.3390/nu12092695
32. Gao M, Wu J, Zhou S, et al. (2024) Combining fecal microbiome and metabolomics reveals diagnostic biomarkers for esophageal squamous cell carcinoma. *Microbiol Spectr* 12: e04012-23. https://doi.org/10.1128/spectrum.04012-23
33. Davis LMG, Martínez I, Walter J, et al. (2011) Barcoded pyrosequencing reveals that consumption of galactooligosaccharides results in a highly specific bifidogenic response in humans. *PLoS ONE* 6: e25200. https://doi.org/10.1371/journal.pone.0025200
34. Raethong N, Nakphaichit M, Suratannon N, et al. (2021) Analysis of human gut microbiome: Taxonomy and metabolic functions in thai adults. *Genes* 12: 331. https://doi.org/10.3390/genes12030331
35. Fernandez-Sanjurjo M, Fernandez J, Martinez-Camblor P, et al. (2024) Dynamics of gut microbiota and short-chain fatty acids during a cycling grand tour are related to exercise performance and modulated by dietary intake. *Nutrients* 16: 661. https://doi.org/10.3390/nu16050661

36. Pinna NK, Anjana RM, Saxena S, et al. (2021) Trans-ethnic gut microbial signatures of prediabetic subjects from India and Denmark. *Genome Med* 13: 36. https://doi.org/10.1186/s13073-021-00851-9
37. Larsen N, Vogensen FK, van den Berg FWJ, et al. (2010) Gut microbiota in human adults with type 2 diabetes differs from non-diabetic adults. *PLoS ONE* 5. https://doi.org/10.1371/journal.pone.0009085
38. Zheng P, Zeng B, Zhou C, et al. (2016) Gut microbiome remodeling induces depressive-like behaviors through a pathway mediated by the host's metabolism. *Mol Psychiatry* 21: 786–796. https://doi.org/10.1038/mp.2016.44
39. González-Zancada N, Redondo-Useros N, Díaz LE, et al. (2020) Association of moderate beer consumption with the gut microbiota and SCFA of healthy adults. *Molecules* 25: 4772. https://doi.org/10.3390/molecules25204772
40. Zhang P, Fang J, Li G, et al. (2021) Sex differences in fecal microbiota correlation with physiological and biochemical indices associated with end-stage renal disease caused by immunoglobulin a nephropathy or diabetes. *Front Microbiol* 12: 752393. https://doi.org/10.3389/fmicb.2021.752393
41. Song ST, Cai LY, Zeng X, et al. (2022) Gut microbial profile in asymptomatic gallstones. *Front Microbiol* 13: 882265. https://doi.org/10.3389/fmicb.2022.882265
42. Volokh O, Klimenko N, Berezhnaya Y, et al. (2019) Human gut microbiome response induced by fermented dairy product intake in healthy volunteers. *Nutrients* 11: 547. https://doi.org/10.3390/nu11030547
43. Zhuang X, Tian Z, Li L, et al. (2018) Fecal microbiota alterations associated with diarrhea-predominant irritable bowel syndrome. *Front Microbiol* 9: 1600. https://doi.org/10.3389/fmicb.2018.01600
44. Blatchford P, Stoklosinski H, Eady S, et al. (2017) Consumption of kiwifruit capsules increases *Faecalibacterium prausnitzii* abundance in functionally constipated individuals: A randomised controlled human trial. *J Nutr Sci* 6: e52. https://doi.org/10.1017/jns.2017.52
45. Sun XZ, Zhao DY, Zhou YC, et al. (2020) Alteration of fecal tryptophan metabolism correlates with shifted microbiota and may be involved in pathogenesis of colorectal cancer. *World J Gastroenterol* 26. https://doi.org/10.3748/wjg.v26.i45.7173
46. McLellan SL, Huse SM, Mueller-Spitz SR, et al. (2010) Diversity and population structure of sewage-derived microorganisms in wastewater treatment plant influent. *Environ Microbiol* 12: 1376. https://doi.org/10.1111/j.1462-2920.2010.02204.x
47. Shen Y, Xu J, Li Z, et al. (2018) Analysis of gut microbiota diversity and auxiliary diagnosis as a biomarker in patients with schizophrenia: A cross-sectional study. *Schizophr Res* 197: 470–477. https://doi.org/10.1016/j.schres.2018.01.002
48. Ruiz-Saavedra S, Zapico A, Del Rey CG, et al. (2022) Dietary xenobiotics derived from food processing: Association with fecal mutagenicity and gut mucosal damage. *Nutrients* 14: 3482. https://doi.org/10.3390/nu14173482
49. Nam YD, Jung MJ, Roh SW, et al. (2011) Comparative analysis of Korean human gut microbiota by barcoded pyrosequencing. *PLoS ONE* 6: e22109. https://doi.org/10.1371/journal.pone.0022109
50. Bjørkhaug ST, Aanes H, Neupane SP, et al. (2019) Characterization of gut microbiota composition and functions in patients with chronic alcohol overconsumption. *Gut Microbes* 10: 663–675. https://doi.org/10.1080/19490976.2019.1580097

51. Zhang YK, Zhang Q, Wang YL, et al. (2021) A Comparison study of age and colorectal cancer-related gut bacteria. *Front Cell Infect Microbiol* 11: 606490. https://doi.org/10.3389/fcimb.2021.606490
52. Hansen TH, Thomassen MT, Madsen ML, et al. (2018) The effect of drinking water pH on the human gut microbiota and glucose regulation: Results of a randomized controlled cross-over intervention. *Sci Rep* 8: 16626. https://doi.org/10.1038/s41598-018-34761-5
53. Zhang Z, Zhu L, Ma Y, et al. (2022) Study on the characteristics of intestinal flora composition in gastric cancer patients and healthy people in the Qinghai-Tibet Plateau. *Appl Biochem Biotechnol* 194: 1510–1526. https://doi.org/10.1007/s12010-021-03732-4
54. Feng Y, Duan Y, Xu Z, et al. (2019) An examination of data from the American Gut Project reveals that the dominance of the genus *Bifidobacterium* is associated with the diversity and robustness of the gut microbiota. *MicrobiologyOpen* 8: e939. https://doi.org/10.1002/mbo3.939
55. Senina A, Markelova M, Khusnutdinova D, et al. (2024) Two-year study on the intra-individual dynamics of gut microbiota and short-chain fatty acids profiles in healthy adults. *Microorganisms* 12: 1712. https://doi.org/10.3390/microorganisms12081712
56. Sugurmar ANK, Mohd R, Shah SA, et al. (2021) Gut microbiota in immunoglobulin a nephropathy: A Malaysian perspective. *BMC Nephrol* 22: 145. https://doi.org/10.1186/s12882-021-02315-z
57. Fontana A, Manchia M, Panebianco C, et al. (2020) Exploring the role of gut microbiota in major depressive disorder and in treatment resistance to antidepressants. *Biomedicines* 8: 311. https://doi.org/10.3390/biomedicines8090311
58. Jiao B, Cao X, Zhang C, et al. (2023) Alterations of the gut microbiota in patients with postherpetic neuralgia. *AMB Express* 13: 108. https://doi.org/10.1186/s13568-023-01614-y
59. Cano-Ortiz A, Laborda-Illanes A, Plaza-Andrades I, et al. (2020) Connection between the gut microbiome, systemic inflammation, gut permeability and FOXP3 expression in patients with primary Sjögren's syndrome. *Int J Mol Sci* 21: 8733. https://doi.org/10.3390/ijms21228733
60. Li F, Wang M, Wang J, et al. (2019) Alterations to the gut microbiota and their correlation with inflammatory factors in chronic kidney disease. *Front Cell Infect Microbiol* 9: 206. https://doi.org/10.3389/fcimb.2019.00206
61. Ceccarani C, Bassanini G, Montanari C, et al. (2020) Proteobacteria overgrowth and butyrate-producing taxa depletion in the gut microbiota of glycogen storage disease type 1 patients. *Metabolites* 10: 133. https://doi.org/10.3390/metabo10040133
62. Yoon H, Park S, Jun YK, et al. (2023) Evaluation of bacterial and fungal biomarkers for differentiation and prognosis of patients with inflammatory bowel disease. *Microorganisms* 11: 2882. https://doi.org/10.3390/microorganisms11122882
63. Rodríguez-Díaz C, Martín-Reyes F, Taminiau B, et al. (2023) The metagenomic composition and effects of fecal-microbe-derived extracellular vesicles on intestinal permeability depend on the patient's disease. *Int J Mol Sci* 24: 4971. https://doi.org/10.1080/19490976.2020.1736974
64. Hou Q, Zhao F, Liu W, et al. (2020) Probiotic-directed modulation of gut microbiota is basal microbiome dependent. *Gut Microbes* 12. https://doi.org/10.1080/19490976.2020.1736974
65. Sisti D, Pazienza V, Piccini F, et al. (2022) A proposal for the reference intervals of the Italian microbiota "scaffold" in healthy adults. *Sci Rep* 12: 3952. https://doi.org/10.1038/s41598-022-08000-x

66. Kalyana Chakravarthy S, Jayasudha R, Ranjith K, et al. (2018) Alterations in the gut bacterial microbiome in fungal Keratitis patients. *PLOS ONE* 13: e0199640. https://doi.org/10.1371/journal.pone.0199640
67. Barengolts E, Green SJ, Eisenberg Y, et al. (2018) Gut microbiota varies by opioid use, circulating leptin and oxytocin in African American men with diabetes and high burden of chronic disease. *PLoS ONE* 13. https://doi.org/10.1371/journal.pone.0194171
68. Kim SW, Suda W, Kim S, et al. (2013) Robustness of gut microbiota of healthy adults in response to probiotic intervention revealed by high-throughput pyrosequencing. *DNA Res* 20: 241–253. https://doi.org/10.1093/dnares/dst006
69. Borgo F, Riva A, Benetti A, et al. (2017) Microbiota in anorexia nervosa: The triangle between bacterial species, metabolites and psychological tests. *PLOS ONE* 12. https://doi.org/10.1371/journal.pone.0179739
70. Wang J, Bleich RM, Zarmer S, et al. (2021) Long-read sequencing to interrogate strain-level variation among adherent-invasive Escherichia coli isolated from human intestinal tissue. *PLOS ONE* 16: e0259141. https://doi.org/10.1371/journal.pone.0259141
71. Gallè F, Valeriani F, Cattaruzza MS, et al. (2020) Mediterranean diet, physical activity and gut microbiome composition: A cross-sectional study among healthy young Italian adults. *Nutrients* 12: 2164. https://doi.org/10.3390/nu12072164
72. Kumar S, Mahajan S, Kale D, et al. (2024) Insights into the gut microbiome of vitiligo patients from India. *BMC Microbiol* 24: 440. https://doi.org/10.1186/s12866-024-03529-5
73. Pallav K, Dowd SE, Villafuerte J, et al. (2014) Effects of polysaccharopeptide from *Trametes Versicolor* and amoxicillin on the gut microbiome of healthy volunteers. *Gut Microbes* 5: 458–467. https://doi.org/10.4161/gmic.29558
74. Lee SM, Kim N, Yoon H, et al. (2021) Compositional and functional changes in the gut microbiota in irritable bowel syndrome patients. *Gut Liver* 15: 253–261. https://doi.org/10.5009/gnl19379
75. Santoru ML, Piras C, Murgia A, et al. (2017) Cross sectional evaluation of the gut-microbiome metabolome axis in an Italian cohort of IBD patients. *Sci Rep* 7. https://doi.org/10.1038/s41598-017-10034-5
76. Das T, Jayasudha R, Chakravarthy S, et al. (2021) Alterations in the gut bacterial microbiome in people with type 2 diabetes mellitus and diabetic retinopathy. *Sci Rep* 11. https://doi.org/10.1038/s41598-021-82538-0
77. Zakerska-Banaszak O, Tomczak H, Gabryel M, et al. (2021) Dysbiosis of gut microbiota in Polish patients with ulcerative colitis: A pilot study. *Sci Rep* 11. https://doi.org/10.1038/s41598-021-81628-3
78. Wong VWS, Tse CH, Lam TTY, et al. (2013) Molecular characterization of the fecal microbiota in patients with nonalcoholic steatohepatitis—A longitudinal study. *PLoS ONE* 8. https://doi.org/10.1371/journal.pone.0062885
79. Reimer RA, Soto-Vaca A, Nicolucci AC, et al. (2020) Effect of chicory inulin-type fructan–containing snack bars on the human gut microbiota in low dietary fiber consumers in a randomized crossover trial. *Am J Clin Nutr* 111: 1286–1296. https://doi.org/10.1093/ajcn/nqaa074
80. Liu W, Zhang R, Shu R, et al. (2020) Study of the relationship between microbiome and colorectal cancer susceptibility using 16SrRNA sequencing. *BioMed Res Int* 2020: 7828392. https://doi.org/10.1155/2020/7828392

81. Hiel S, Bindels LB, Pachikian BD, et al. (2019) Effects of a diet based on inulin-rich vegetables on gut health and nutritional behavior in healthy humans. *Am J Clin Nutr* 109: 1683–1695. https://doi.org/10.1093/ajcn/nqz001
82. Yu X, Ge P, Zhai Y, et al. (2023) Gut microbiota in adults with moyamoya disease: Characteristics and biomarker identification. *Front Cell Infect Microbiol* 13: 1252681. https://doi.org/10.3389/fcimb.2023.1252681
83. Yang Y, Misra BB, Liang L, et al. (2019) Integrated microbiome and metabolome analysis reveals a novel interplay between commensal bacteria and metabolites in colorectal cancer. *Theranostics* 9: 4101–4114. https://doi.org/10.7150/thno.35186
84. Yang Y, Du L, Shi D, et al. (2021) Dysbiosis of human gut microbiome in young-onset colorectal cancer. *Nat Commun* 12: 6757. https://doi.org/10.1038/s41467-021-27112-y
85. Yeoh YK, Chen Z, Wong MCS, et al. (2020) Southern Chinese populations harbour non-nucleatum Fusobacteria possessing homologues of the colorectal cancer-associated FadA virulence factor. *Gut* 69: 1998–2007. https://doi.org/10.1136/gutjnl-2019-319635
86. Ren Z, Fan Y, Li A, et al. (2020) Alterations of the human gut microbiome in chronic kidney disease. *Adv Sci* 7: 2001936. https://doi.org/10.1002/advs.202001936
87. van Zanten GC, Krych L, Röytiö H, et al. (2014) Synbiotic *Lactobacillus acidophilus* NCFM and cellobiose does not affect human gut bacterial diversity but increases abundance of lactobacilli, bifidobacteria and branched-chain fatty acids: A randomized, double-blinded cross-over trial. *FEMS Microbiol Ecol* 90: 225–236. https://doi.org/10.1111/1574-6941.12397
88. Gózd-Barszczewska A, Kozioł-Montewka M, Barszczewski P, et al. (2017) Gut microbiome as a biomarker of cardiometabolic disorders. *Ann Agric Environ Med* 24: 416–422. https://doi.org/10.26444/aaem/75456
89. Zhang J, Guo Z, Lim AAQ, et al. (2015) Mongolians core gut microbiota and its correlation with seasonal dietary changes. *Sci Rep* 4: 5001. https://doi.org/10.1038/srep05001
90. Plaza-Díaz J, Fernández-Caballero J, Chueca N, et al. (2015) Pyrosequencing analysis reveals changes in intestinal microbiota of healthy adults who received a daily dose of immunomodulatory probiotic strains. *Nutrients* 7: 3999–4015. https://doi.org/10.3390/nu7063999
91. Lindheim L, Bashir M, Münzker J, et al. (2017) Alterations in gut microbiome composition and barrier function are associated with reproductive and metabolic defects in women with Polycystic Ovary Syndrome (PCOS): A pilot study. *PLOS ONE* 12: e0168390. https://doi.org/10.1371/journal.pone.0168390
92. Clos-Garcia M, Garcia K, Alonso C, et al. (2020) Integrative analysis of fecal metagenomics and metabolomics in colorectal cancer. *Cancers* 12. https://doi.org/10.2139/ssrn.3520024
93. Yoshida N, Watanabe S, Yamasaki H, et al. (2022) Average gut flora in healthy Japanese subjects stratified by age and body mass index. *Biosci Microbiota Food Health* 41: 2021–2056. https://doi.org/10.12938/bmfh.2021-056
94. La-ongkham O, Nakphaichit M, Nakayama J, et al. (2020) Age-related changes in the gut microbiota and the core gut microbiome of healthy Thai humans. *3 Biotech* 10: 276. https://doi.org/10.1007/s13205-020-02265-7
95. Wang T, Cai G, Qiu Y, et al. (2012) Structural segregation of gut microbiota between colorectal cancer patients and healthy volunteers. *ISME J* 6: 320–329. https://doi.org/10.1038/ismej.2011.109

96. Odamaki T, Kato K, Sugahara H, et al. (2016) Age-related changes in gut microbiota composition from newborn to centenarian: A cross-sectional study. *BMC Microbiol* 16: 90. https://doi.org/10.1186/s12866-016-0708-5
97. Mingjun Z, Fei M, Zhousong X, et al. (2022) 16S rDNA sequencing analyzes differences in intestinal flora of human immunodeficiency virus (HIV) patients and association with immune activation. *Bioengineered* 13: 4085–4099. https://doi.org/10.1080/21655979.2021.2019174
98. Zapała B, Stefura T, Wójcik-Pędziwiatr M, et al. (2021) Differences in the composition of gut microbiota between patients with Parkinson's disease and healthy controls: A cohort study. *J Clin Med* 10: 5698. https://doi.org/10.3390/jcm10235698
99. Zhou L, Ni Z, Cheng W, et al. (2020) Characteristic gut microbiota and predicted metabolic functions in women with PCOS. *Endocr Connect* 9: 63–73. https://doi.org/10.1530/EC-19-0522
100. Seong G, Kim N, Joung JG, et al. (2020) Changes in the intestinal microbiota of patients with inflammatory bowel disease with clinical remission during an 8-week infliximab infusion cycle. *Microorganisms* 8: 874. https://doi.org/10.3390/microorganisms8060874
101. Copeland JK, Chao G, Vanderhout S, et al. (2021) The Impact of migration on the gut metagenome of South Asian Canadians. *Gut Microbes* 13: 1902705. https://doi.org/10.1080/19490976.2021.1902705
102. Caleça T, Ribeiro P, Vitorino M, et al. (2023) Breast cancer survivors and healthy women: Could gut microbiota make a difference?—"BiotaCancerSurvivors": A case-control study. *Cancers* 15: 594. https://doi.org/10.3390/cancers15030594
103. Esteban-Torres M, Santamaría L, Cabrera-Rubio R, et al. (2018) A diverse range of human gut bacteria have the potential to metabolize the dietary component gallic acid. *Appl Environ Microbiol* 84. https://doi.org/10.1128/AEM.01558-18
104. Sankarasubramanian J, Ahmad R, Avuthu N, et al. (2020) Gut microbiota and metabolic specificity in ulcerative colitis and crohn's disease. *Front Med* 7: 606298. https://doi.org/10.3389/fmed.2020.606298

AIMS Press

© 2025 the Author(s), licensee AIMS Press. This is an open access article distributed under the terms of the Creative Commons Attribution License (https://creativecommons.org/licenses/by/4.0)
